# Mental Reactivation and Pleasantness Judgment of Experience Related to Vision, Hearing, Skin Sensations, Taste and Olfaction

**DOI:** 10.1371/journal.pone.0159036

**Published:** 2016-07-11

**Authors:** Marina G. Kolbeneva, Yuri I. Alexandrov

**Affiliations:** 1 V.B. Shvyrkov Laboratory of Neuronal Bases of Mind, Institute of Psychology, Russian Academy of Sciences, Moscow, Russian Federation; 2 Department of Psychology, National Research University Higher School of Economics, Moscow, Russian Federation; University of Akron, UNITED STATES

## Abstract

Language acquisition is based on our knowledge about the world and forms through multiple sensory-motor interactions with the environment. We link the properties of individual experience formed at different stages of ontogeny with the phased development of sensory modalities and with the acquisition of words describing the appropriate forms of sensitivity. To test whether early-formed experience related to skin sensations, olfaction and taste differs from later-formed experience related to vision and hearing, we asked Russian-speaking participants to categorize or to assess the pleasantness of experience mentally reactivated by sense-related adjectives found in common dictionaries. It was found that categorizing adjectives in relation to vision, hearing and skin sensations took longer than categorizing adjectives in relation to olfaction and taste. In addition, experience described by adjectives predominantly related to vision, hearing and skin sensations took more time for the pleasantness judgment and generated less intense emotions than that described by adjectives predominantly related to olfaction and taste. Interestingly the dynamics of skin resistance corresponded to the intensity and pleasantness of reported emotions. We also found that sense-related experience described by early-acquired adjectives took less time for the pleasantness judgment and generated more intense and more positive emotions than that described by later-acquired adjectives. Correlations were found between the time of the pleasantness judgment of experience, intensity and pleasantness of reported emotions, age of acquisition, frequency, imageability and length of sense-related adjectives. All in all these findings support the hypothesis that early-formed experience is less differentiated than later-formed experience.

## Introduction

Our knowledge forms through multiple sensory-motor interactions with the environment. As part of individual development, language acquisition can be considered as learning to achieve goals through verbal and nonverbal communication with other individuals [1]. If so, all knowledge can be viewed as a set of internal models of individual interactions with the environment. Although another position suggests that knowledge is stored in the form of symbols [2], the idea of a strong connection between language and interactions has been consistent and is attracting more experimental support [3–12], as are the theories that knowledge is embodied (see, e.g., [13–15]).

Nevertheless, there is no recent empirical support for strongly embodied and completely disembodied theories [16]. Therefore, an important problem requiring further investigation is the degree to which conceptual representations are grounded in sensory and motor processes [17]. The contribution of each modality in different categories of objects has been studied using the methods of multi-modal rating of objects [18]. These data demonstrated that the majority of objects’ characteristics were rated as visual. Smell and taste were relevant for a minority of objects, whereas tactile sensations and sound occupied the intermediate positions. Similar results were obtained for concept-property items when participants rated the degree to which the property could be experienced through five perceptual modalities (vision, audition, touch, smell, and taste) [19–20]. However, the specificity of experience related to various senses is still unclear. This study is aimed to analyse the peculiarities of the structure of experience related to different senses through the mental reactivation of experience and its pleasantness judgment.

As an individual interacts with the environment the models of such interactions accumulate in the structure of individual experience so that during development the structure of experience becomes more complex and differentiated. Numerous experiments with neural activity recording in animals and humans, ([21–31]; and others) show that learning requires the specialization of distributed networks of neurons. Such networks of neurons along with other morphological elements constituting the rest of the body, comprise functional systems (internal models; [21], [26], [29]). Their activity underlies behavioral acts aimed at achieving adaptive results. Formation of new functional systems during development results in growing complexity and differentiation of organism-environment relations [32–36]. Ontogenetic development can be considered as the process of increasing differentiation along with the number of learnt behaviours (see [26], [32], [36–42]). Studies show that in mammalian embryogenesis, skin sensitivity and probably taste and olfaction start to develop earlier than hearing and vision [43–44]. Based on these data, we hypothesized that early-formed experience predominantly related to skin sensations, olfaction and taste is less differentiated than later-formed experience predominantly related to hearing and vision.

The differentiation of experience related to senses can be analysed in several ways. In our opinion, the quantity of words available lexicographically (i.e., as found in common dictionaries) and used to describe behaviour may indicate the degree of differentiation of sense-related experience. Slobin [45] notes that children acquire a well-developed vocabulary to report sensations received through vision and hearing. However, vocabulary reporting proximal sensations (smell, taste, touch) is inadequate. Chernigovskaya and Arshavsky [46] also find that the visual semiosphere is probably the most thoroughly elaborated by the majority of human languages, whereas the olfactory semiosphere is the least verbalized of all sensory modalities. It was found in English, Japanese and Zulu that two-thirds or even three-quarters of the words describing sensations refer to vision and hearing, and only a minor part of the remaining words was distributed among all of the other senses [47]. In this work, we evaluated the distribution of Russian adjectives describing different sensations in accordance with participants’ answers.

The choice of adjectives was made because adjectives do not describe a whole object, but only its properties. Adjectives describe the different properties of an object as perceived by different senses. In contrast, nouns describe concrete objects and always invite a relatively multimodal simulation [48]. Adjectives describing sensations relate to a valence of emotions, as shown previously using a semantic differential measurement technique [49]. Data show that the grammatical category of a noun is established earlier than other grammatical categories and that the acquisition of the other grammatical categories, including adjectives, depends crucially on the prior acquisition of nouns (see, e.g., [50]). Thus, it can be assumed that the processes of differentiation of individual experience are most fully reflected in the characteristics of adjectives.

Another way to assess the differentiation of experience related to senses is through the speed of the mental reactivation of experience using of words. The more differentiated the experience is, the more functional systems are reactivated, which may require more time. Considering that experience related to early-developed senses is less differentiated than experience related to later-developed senses, the former should be mentally reactivated faster than the latter. Differences in the reactivation time of early- and later-developed experience were studied in relation to the age of (word) acquisition (AoA). It is shown that early-acquired words are recalled, read and recognized faster than are later-acquired words [51–54]. Therefore, we compared the time of categorization and pleasantness judgment of experience related to early- and later-formed senses and experience that is mentally reactivated by means of early- and later-acquired adjectives.

We also think that the differentiation of experience related to senses can be manifested through subjective evaluation of that experience. It has been shown previously that intense emotions characterize the reactivation of low-differentiated functional systems formed at the early stages of individual development [55–58]. Therefore, in this work, we relate emotion to the most ancient, low-differentiated levels of the organization of behaviour (for related views, see [27], [37], [59–64]). We compared the intensity of emotions reported by participants and galvanic skin responses as an objective indicator of emotional involvement [65–66] during an assessment of experience on the pleasantness scale.

It has been shown that the emotional connotation of words can influence both the processing of those words (see, e.g., [67]) and the re-enactment of movements related to them [68–69]. In this work, we compared the time of mental reactivation of sense-related experience rated as causing more or less pleasant and unpleasant emotions.

In summary, our study links the properties of individual experience formed at different stages of ontogeny both with the phased development of sensory modalities upon which this experience is based and with the acquisition of words describing the appropriate forms of sensitivity.

## Material and Methods

### 1.1. Ethical Statement

All of the participants gave written informed consent to take part in the study after receiving an explanation of the procedures. We did not obtain informed consent for any of the experiments from the next of kin, caretakers, or guardians on behalf of the 15-17-year-old participants enrolled in our study because in the Russian Federation, those participants have a right to give their own written informed consent in this situation. The Ethics Committee of Federal State-Financed Institution, Institute of Psychology, Russian Academy of Sciences, Moscow approved the experimental protocol of our study, approved the specific consent procedure for minors and assessed our study as safe for the participants’ psychic and physical health.

### 1.2. Subjects

Computerized categorization of adjectives (Experiment 1) was done by a sample of 115 native Russian-speaking psychology students (79 women, aged 17 to 36, median age 19) and by an additional sample of 13 students (10 women, aged from 17 to 23, median age 18). Each participant received payment (the equivalent of $5 in roubles).

Another sample of 97 native Russian-speaking psychology students (70 women, aged 15 to 26, median age 18) assessed pleasantness of experience as mentally reactivated through sense-related adjectives (Experiment 2). There was only one 15-year-old participant (university student) and no 16-year-old participants in the study. Each participant received payment (the equivalent of $5 in roubles). Skin resistance was measured in 23 out of 97 participants (14 women, aged 17 to 25, median age 18).

### 1.3. Materials

The initial categorization of adjectives was done by 44 native Russian-speaking psychology students (23 women, aged 17 to 28, median age 18). All of the adjectives (15,918 in total) listed in the Russian dictionary [70] were printed in random order on 94 worksheets. Participants received these worksheets with adjectives and a sheet with instructions in Russian, which translate into English as follows: “Please mark with a cross the box next to those adjectives that do not describe any of sensations received through eyes, ears, nose, tongue, hands, body, and sensations of internal organs. Do not dwell on the answer: trust your first impression. If the adjective is not familiar to you, mark it with the letter ‘n’ in the box next to it”. The participants performed the task at their leisure over a month.

Using binomial m criteria, we tested whether the frequency of answers “not related” and “not familiar” for each adjective was greater than 50% probability. The results showed that 8,302 adjectives were classified by 31 or more participants as either unrelated to senses or unknown (p < .01) and were excluded from further research. The remaining 7,616 adjectives were used for computerized categorization.

#### Experiment 1. Computerized categorization of adjectives into different senses

The adjectives (7,616 overall) selected in the course of the initial categorization were randomly divided into three lists of 2,538, 2,539 and 2,539 adjectives. Each participant rated only one list of adjectives during the computerized categorization. An additional sample of 13 participants received all three lists of adjectives and performed the task in several sessions (one session per day).

#### Experiment 2. Pleasantness judgment of experience mentally reactivated by means of sense-related adjectives

Based on the results of Experiment 1, we selected 475 adjectives (data are available in the S1 Appendix). During this selection, we aimed to include the maximum possible number of different multimodal adjectives. These 475 adjectives included 34 vision-related, 111 hearing-related, 71 skin sensitivity-related, 61 taste-related, 64 olfaction-related, 9 vision and hearing-related, 49 vision and skin sensitivity-related, 21 vision and taste-related, 18 vision and olfaction-related, 20 taste and olfaction-related, and 18 vision and taste and olfaction-related adjectives. The resulting lists contained 120 adjectives related to one of the senses, i.e., vision, hearing, skin sensitivity, olfaction and taste (Table 1). Properties of each sense-related adjective see in S1 Appendix. We also introduced 25 additional adjectives (5 adjectives for each of the senses) to use in training before each session.

**Table 1:** Properties of sense-related adjectives used for the mental reactivation of experience.

Sense-related adjectives	Lexical frequency among 192,689,044 wordforms, mean (median)	Adjectives with unknown lexical frequency, number	Letter, mean (median)	Syllable, mean (median)	Imageability (rank), mean (median)	AoA (rank), mean (median)	Number of the adjectives with different AoA among sense-related adjectives						
							0–2 years	3–4 years	5–6 years	7–8 years	9–10 years	11–12 years	13-… years
**Vision** (N = 120)	198.96 (36)	12	8.79 (9)	3.30 (3)	28.17 (29)	3.07 (3)	0	28	62	24	6	0	0
**Hearing** (N = 120)	215.22 (48)	17	10.24 (10)	3.88 (4)	25.34 (28)	3.78 (4)	0	9	41	44	20	6	0
**Skin sensations** (N = 120)	184.24 (40.5)	20	9.15 (9)	3.34 (3)	27.30 (29)	3.56 (3.5)	0	11	49	42	18	0	0
**Taste** (N = 120)	143.18 (19.5)	18	8.98 (9)	3.35 (3)	25.80 (28)	2.99 (3)	0	39	51	23	6	1	0
**Olfaction** (N = 120)	184.98 (35.5)	16	8.68 (9)	3.33 (3)	24.48 (27)	3.33 (3)	0	29	45	30	11	4	1

Frequency data were taken from the Russian national corpus [71]. The data available from the Russian national corpus included frequency for only those words that occur at least 3 times among 192,689,044 wordforms; such data were not available for 16% of our adjectives (see Table 1). These 16% of adjectives were assigned a frequency equal to 1 because all of them had been taken from the common dictionary of Russian language [70] and therefore occurred at least once. There was no significant difference in the frequency between the lists of adjectives related to vision, hearing, olfaction, taste and tactile sensations (Kruskal-Wallis H, χ^2^ = 3.530, df = 4, p = .473).

Significant differences were revealed in the length and number of syllables between the lists of adjectives (Kruskal-Wallis H, χ^2^ = 25.17, df = 4, p < .001; χ^2^ = 19.62, df = 4, p = .001, correspondingly). Subsequent pairwise comparisons with a Mann-Whitney U test revealed that only hearing-related adjectives were longer and included a greater number of syllables compared with all other sense-related adjectives. The rest of the sense-related adjectives did not differ from each other (see Table 1).

#### AoA of the adjectives related to different senses

An independent sample of 107 native Russian-speaking students (87 women, aged 18 to 32, median age 19) reported the AoA of 475 adjectives relating to different senses (vision, hearing, skin sensitivity, olfaction and taste) as used in Experiment 2 for the mental reactivation of experience (see Table 1). To define the AoA of adjectives, we used a modified method of subjective rating of AoA [72]. Adjectives, regardless of their relationship to senses, were printed in random order in two columns on a single page. Participants were asked to choose the approximate age when they learned each of the words on the page from several presented age intervals (detailed description of the method see in S2 Appendix). No time limit was placed on responses. The experiment lasted 15–20 minutes. Participants received one page of adjectives per week (five pages in total).

Not all of the participants rated all five lists of the adjectives. Every adjective was presented to 76–85 participants. Responses were placed on an 8-point scale (from 1 for age 0 to 2, to 7 for age 13 or older, and 8 for unknown adjectives). The median value of AoA was computed for every adjective (the percentage of adjectives with different AoA among adjectives related to different senses see in Table 1). Because of the very small number of adjectives with AoA in the range of 11 years and older, those adjectives were pooled with the adjectives with AoA in the range between 9 and 10 years in all computations.

#### Imageability of sense-related adjectives

An independent sample of 60 native Russian-speaking students (52 women, aged 17 to 33, median age 18) rated the imageability of 475 adjectives related to various senses as used in Experiment 2 (see Table 1). Because some adjectives described more than one type of sensation, they were presented more than once, extending the total number of word presentations to 600. The adjectives comprised 5 worksheets containing 2 columns of 120 randomly printed adjectives, relating to one of the senses. One of the following words was printed to the right of each adjective: “object” for adjectives describing visual or skin sensations, “sound” for adjectives describing auditory sensations, “taste” for adjectives describing taste sensations, and “smell” for adjectives describing olfactory sensations. There was an empty box to the right of each word combination. A question was printed at the top of each worksheet. For adjectives related to vision, the question was: “Can I imagine what I see?” For adjectives describing auditory, tactile, taste and olfactory sensations, the verb “see” was changed to “hear”, “touch”, “feel”, or “perceive”.

Participants received one worksheet of adjectives and were asked to silently ask themselves the question for each word combination and mark those adjectives that described something they could not imagine. No time limit was placed on responses. After a participant had responded to all of the word combinations on the worksheet, she received the next one (five overall). The experiment lasted 30–40 minutes. The order of the five worksheets was counterbalanced in a Latin square format for different participants. The imageability of adjectives was determined by ranking participants’ responses. A maximal rank (imageability) was assigned to adjectives with the smallest number of participants’ marks.

#### Additional categorization of adjectives related to skin sensitivity

To test the assumption that adjectives related to skin sensations are heterogeneous, we presented an independent sample of 62 native Russian-speaking psychology students (50 women, aged 17 to 33, median age 18) with 120 adjectives related to skin sensitivity (used in Experiment 2 for the mental reactivation of experience) for additional categorization. Adjectives were printed on a blank in a random order in two columns. On the right of each column of the adjectives there were two blank columns headed with Cyrillic letters “T” and “P”. The letter “T” stood for the first letter of the word “тело” (“body” in English), letter “P” stood for the first letter of the word “рука” (“hand” in English). There were two blank boxes next to each adjective. Participants were asked to mark those boxes which corresponded to how they usually perceive the characteristic denoted by each of the adjectives on the page (detailed description of the method see in S2 Appendix). The experiment lasted 20 minutes. Those adjectives with more than 32 isolated ticks in the “hand” column were rated as touch-related (54 adjectives in total, χ^2^ (1, N = 62) = 4.22, p < .05). There was no adjectives with more than 32 isolated ticks in the “body” column. The sum of isolated ticks in the “body” column and double ticks were counted for each adjective. Adjectives with more than 51 such responses overall were rated as related to body skin sensations (17 adjectives in total, χ^2^ (1, N = 62) = 4.05, p < .05). Other adjectives were rated as related to mixed skin sensations (49 adjectives in total) (data are available in the S1 Appendix).

### 1.4. Experiment 1 (categorization of adjectives to different senses)

Experiment 1 aimed to verify the following hypotheses: (1) the quantity of adjectives used to describe visual sensations and sounds that can be found in dictionaries is greater than the quantity of adjectives used to describe tactile, gustatory and olfactory sensations; and (2) it takes more time to categorize adjectives in relation to visual sensations and sounds than it takes to categorize adjectives in relation to tactile, gustatory and olfactory sensations.

Each participant was seated in a quiet testing room approximately 50 cm from the computer display. We used a custom experiment software. The software was written for Microsoft Windows XP in C++ with the use of DirectX. DirectInput was used to achieve minimum key-press time measurement delays and deviation. The source code archive of the experiment software is attached in the supplementary materials (S1 Code).

**Fig 1:**
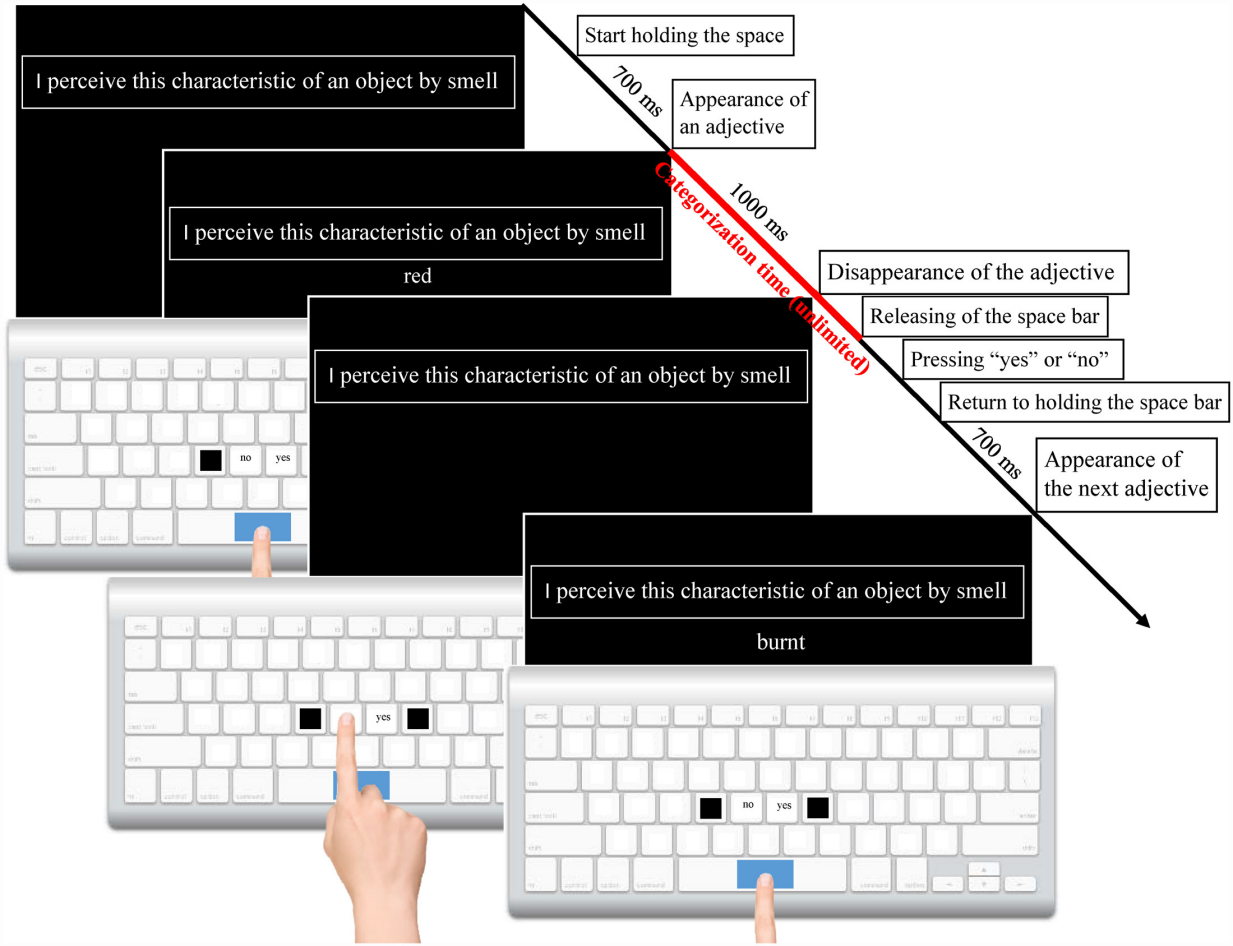
Schematic representation of computerized categorization of adjectives (Experiment 1). A standard white keyboard for Windows (qwerty) was used, with the “G” and “H” keys labelled as “нет” and “да” (Russian equivalent of “no/not” and “yes”, Times New Roman, 16, bold). A separate group of 36 female participants performed the task with the keys for “no/not” and “yes” placed in the reverse order. The “F” and “J” keys were removed to prevent participants from pressing the wrong keys. The central part of the space bar was covered with 3 cm of blue tape. The other keys were covered with white, non-transparent sticky tape. Each session began with the presentation of a sense-related sentence. We used 5 sense-related sentences, translating into English as follows: “I perceive this characteristic of an object by vision” (hearing, smell, taste or touch). Participants were asked to press and hold the space bar on a keyboard. Adjectives were presented one at a time for 1000 ms underneath a sense-related sentence. Participants were asked to press the key labelled “yes” if they agreed or “no/not” if they disagreed that the presented adjective describes what that can be perceived in the sensory modality featured in the sense-related sentence. They were asked to wait for an adjective to disappear before giving an answer. To give an answer, participants had to stop pressing the space bar and press either the key labelled “yes” or the key labelled “no/not”; they were then required to return their index fingers to the central (coloured) part of the space bar. The next adjective appeared 700 ms after participants resumed pressing the space bar.

Participants were briefly familiarized with the apparatus, then they read the instructions. The main study consisted of five blocks. Each block contained five sessions. (see Fig 1).

The procedure allowed us to measure the time required to categorize each adjective: the interval between appearance of an adjective on the screen and releasing the space bar. In the course of one session, 104 or 105 adjectives were successively presented underneath one of the sense-related sentences (the first three presented adjectives were considered as training). Next, the sentence was changed and a new session was started. All five sense-related sentences were presented only once in each block. Participants took a rest break after each block. The experiment lasted 3–4 hours.

The order of the first three training adjectives was the same for all participants. The experimental adjectives (2,538 or 2,539 adjectives) were randomly divided into 25 session lists. The adjectives within each session list were presented in a pseudorandom order allowing a random presentation of the entire set without repetition. The combination of the session lists of adjectives and sense-related sentences was counterbalanced across subjects (for example, one session list of adjectives was presented under a vision-related sentence for one participant and under a hearing-related sentence for another participant, etc.). Consequently, every adjective was assessed by 9–13 participants underneath each sense-related sentence.

### 1.5. Experiment 2 (pleasantness judgment of sense-related experience)

Experiment 2 was designed to verify eight hypotheses: (1) the emotions reported during the mental reactivation of experience related to vision, hearing and touch are less intense than the emotions reported during the mental reactivation of experience related to taste, olfaction and body skin sensations; (2) the higher intensity and higher positivity of emotions reported during mental reactivation of sense-related experience is accompanied by higher frequency, shorter latency, higher amplitude and longer duration of skin-resistance falls; (3) the pleasantness judgment of experience related to vision, hearing and touch takes more time than the pleasantness judgment of experience related to taste, olfaction and body skin sensations; (4) the time of the pleasantness judgment negatively correlates with the intensity and pleasantness of emotions reported during mental reactivation of sense-related experience; (5) later AoA of the adjectives corresponds to a greater time of the pleasantness judgment of experience mentally reactivated by means of these adjectives; (6) participants report less intense and more negative emotions during the mental reactivation of experience by means of later-acquired adjectives than by means of early-acquired adjectives; and (7) characteristics of sense-related adjectives correlate with characteristics of the pleasantness judgment of sense-related experience.

Participants were seated in a quiet testing room approximately 50 cm from the computer display. We used the same custom software as in Experiment 1. Each participant was given a 5-minute training session at the beginning and after the third and the fifth blocks of the study. In the course of the training sessions, participants learnt to use the keyboard in a specific manner, and we received individual timing data about simple decision making for every response key.

**Fig 2:**
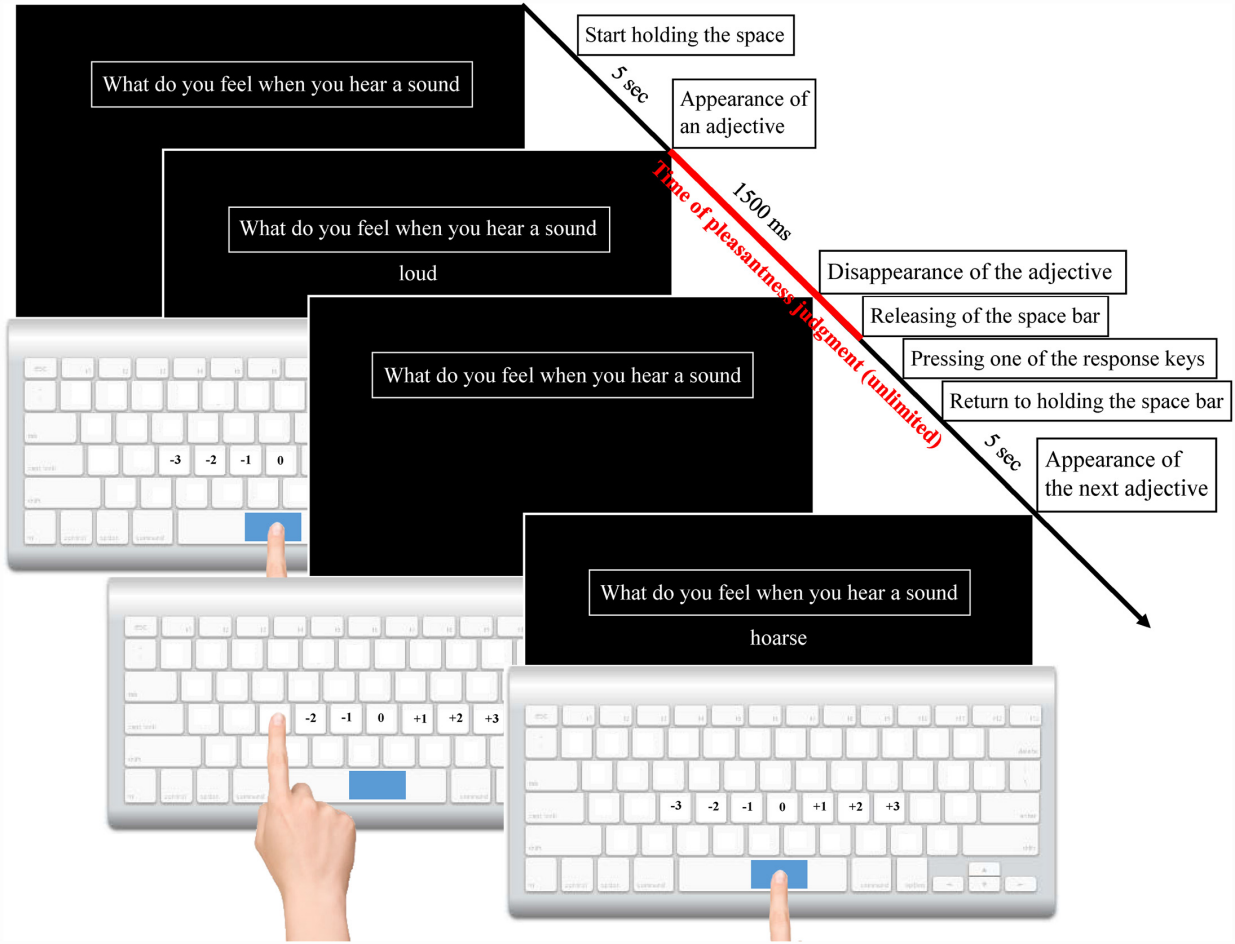
Schematic representation of the pleasantness judgment of experience mentally reactivated by means of sense-related adjectives (Experiment 2). The keys “D”, “F”, “G”, “H”, “J”, “K”, and “L” were labelled, respectively, as “-3”, “-2”, “-1”, “0”, “+1”, “+2”, and “+3” (Times New Roman, 18). The appropriate sense-related adjectives were presented one at a time for 1500 ms underneath the sense-related question. We used 5 sense-related questions for the mental reactivation of experience, translating into English as follows: “What do you feel when you see an object” (hear a sound, sense a smell, sense a taste or touch an object). Participants were asked to imagine the behaviour described in the question that was completed with each of the presented adjectives. Next, the participants had to assess the un/pleasantness of generated emotions by pressing one of the seven response keys on the keyboard, with very pleasant at +3, very unpleasant at -3 and neutral at 0. After that, participants had to move their index fingers to hold down the space bar. Participants were not time-limited when assessing their emotions but they were advised not to spend much time thinking about their response because the “first feelings” are usually the most reliable and of prime interest in this study. The participants were asked to wait for the adjective to disappear before giving an answer. The next adjective appeared 5 seconds after participants resumed pressing the space bar.

Prior to the experimental sessions, each participant received oral instructions from a researcher and read a brief instruction in Russian on the computer screen. The procedure was the same as in Experiment 1 with some differences in instruction and time intervals (see Fig 2).

This technique allowed us to measure the time required for the pleasantness judgment of experience described by means of each adjective.

The study consisted of five blocks. Each block contained five sessions. In the course of the session, 25 appropriate adjectives were successively presented (the first adjective was training). Next, the question was changed and a new session started. All five questions were presented once for each block.

Experimental adjectives were presented in random order without recurrence underneath the corresponding sense-related questions. The presentation order was independently randomized for each participant. Multisensory adjectives were presented once under each corresponding question. The order of training adjectives was the same for all participants. The order of presentation of five questions through five blocks was counterbalanced in a Latin square format. Thus, over the course of the study 625 adjectives (120 adjectives for each sense and 25 training adjectives) were presented. Participants took a rest break after each block. The experiment lasted 3–4 hours.

Skin resistance was measured in 23 out of 97 participants of Experiment 2. Skin-resistance sensors were attached to the fingers of each participant’s non-dominant hand at the beginning of the study. Participants were asked not to move the non-dominant hand, keeping it on their knee, and to try not to take deep breaths during the study. The room temperature was 21–24°C. Skin-resistance responses were recorded with an outside source of voltage (1 V) [73]. Two Cu electrodes shaped as semi rings (contact area 1 cm^2^) were attached without electrolytes to the distal phalanxes of the index and ring fingers. All of the recordings were performed using ADC-based equipment manufactured by Neurocom (Moscow). The sampling frequency was 8 Hz. The accuracy resolution for amplitude was 10 Ohms.

### 1.6. Data analysis

Distributions of values of categorization time, pleasantness ratings, characteristics of skin-resistance falls and time of the pleasantness judgment differed from the normal distribution; therefore, we calculated medians and interquartile ranges. For this reason, we also used nonparametric tests to compare distributions (Wilcoxon signed-rank test, Spearman’s Rho correlation, etc.). Values of the time of the pleasantness judgment were normalized using Tukey normalization (in SPSS) before applying repeated-measures analysis (variant of ANOVA). An alpha level of .05 (p two-tailed) was used for all statistical tests.

In Experiment 1, an independent variable was a sense-related sentence. Dependent variables were the type of answer (yes and no/not) and the time of categorization (in ms).

In Experiment 2, the sense to which the adjectives related was an independent variable. Pleasantness ratings were used as two dependent variables: valence of emotions (+/-), and intensity of emotions, ranging from 0 to 3. Other dependent variables were time of the pleasantness judgment (in ms), frequency, latency, amplitude and duration of skin-resistance falls, AoA and imageability of sense-related adjectives. The length, number of syllables and adjective frequency were controlled variables.

Falls in skin resistance were analysed if they occurred with a latency not less than 125 ms following the appearance of an adjective, with a duration from the beginning of the fall to its lowest point of not less than 500 ms. For each trial of the mental reactivation of experience using a sense-related question completed by one of the adjectives, we analysed only one skin resistance fall (with greatest amplitude) occurring in the interval between the appearance of this adjective and the appearance of the next adjective. Skin resistance analysis included calculation of the latency of skin-resistance falls, amplitude (difference between skin resistance values at the beginning of skin resistance fall and at its lowest point) and duration of skin resistance fall (from the beginning of skin resistance fall to its lowest point). We calculated the median values of latency, amplitude and duration of skin-resistance falls for each participant within those groups of trials, which were assessed with the same rating on a pleasantness scale. This was done separately for different senses. An index of skin-resistance falls frequency was calculated for each participant as a ratio of the quantity of trials in which skin-resistance falls occurred to the total number of trials.

## Results

### 2.1. The quantity of adjectives related to different senses

The sense to which an adjective predominantly relates was determined statistically by tallying the differences between the number of “yes” and “no/not” responses for each adjective in relation to each of the sense-related sentences (quantities of categorized adjectives see in Table 2).

**Table 2:** Distribution of adjectives related to different senses. Using binomial m criteria, we tested whether the frequency of the answer “yes” for each adjective was greater than 50%. Numbers without parentheses indicate quantity of adjectives related to a sense at p = .01 (*m* binomial test, P = 0.50), numbers in parentheses—at p = .05.

Type of adjectives	Vision	Taste	Olfaction	Skin sensations	Hearing	Total number
**Adjectives related only to one sense**	867 (1800)	100 (131)	66 (96)	58 (101)	44 (115)	1135 (2243)
**Adjectives related to two senses**	Vision and Skin, 28 (125)	Taste and Skin, 2 (2)	Olfaction and Skin, 0 (1)	Vision and Skin, 28 (125)	Vision and Hearing, 3 (9)	74 (283)
	Vision and Taste, 8 (22)	Vision and Taste, 8 (22)	Olfaction and Taste, 29 (88)	Taste and Skin, 2 (2)		
	Vision and Olfaction, 2 (18)	Olfaction and Taste, 29 (88)	Vision and Olfaction, 2 (18)	Olfaction and Skin, 0 (1)		
	Vision and Hearing, 3 (9)					
**Adjectives related to three senses**	Vision and Olfaction and Taste, 2 (20)	Vision and Olfaction and Taste, 2 (20)	Vision and Olfaction and Taste, 2 (20)	Vision and Olfaction and Skin, 0 (1)	0	2 (21)
**Total number of adjectives**	910 (1994)	141 (263)	99 (223)	88 (230)	47 (124)	1211 (2547)

As shown in Table 2, vision-related adjectives predominate over adjectives describing all of the other types of sensation listed lexically in the Russian dictionary. This result is only partially consistent with our hypothesis: contrary to the hypothesis, the quantity of hearing-related adjectives was the smallest among sense-related adjectives. There was a high quantity of bimodal visual-haptic and olfactory-gustatory adjectives, whereas the lowest quantity of bimodal adjectives was related to hearing. It should also be noted that only one third of the adjectives (2,547 out of 7,616) presented to participants were categorized by them as related to one or another sense at statistically significant level.

### 2.2. Time of the categorization of adjectives in relation to different senses

**Fig 3:**
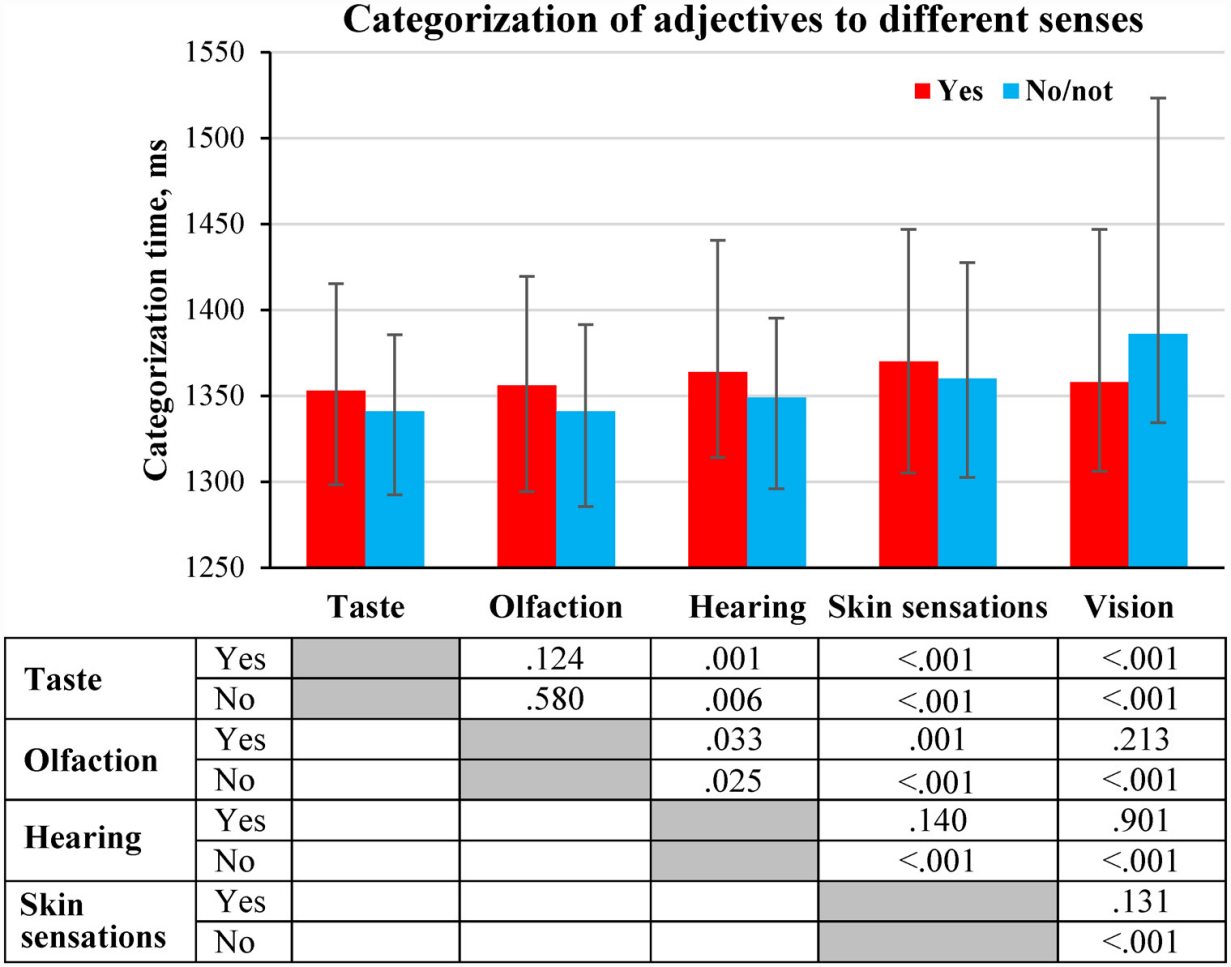
Time of categorization of adjectives to different senses (medians and interquartile ranges). Median values of time of categorization were calculated for each participant for answers “yes” and “no/not”; this was done separately for groups of adjectives presented with different sense-related sentences (see S1 Data). The distributions of the medians of categorization time were compared using Wilcoxon signed-rank test (N = 115). The statistical significance of the differences is displayed in the table under the figure. Here and in other figures it might appear that the height of the bars does not always directly reflect significance of the revealed differences. Please, note that we compared the entire distributions of variables within subjects.

We compared time that participants took to categorize adjectives in relation to different senses (Fig 3).

Fig 3 demonstrates that the categorization of adjectives as related to vision, hearing or skin sensations (answers “yes”), required more time than the categorization of adjectives as related to taste or olfaction (but difference between vision and olfaction was not significant). This result is only partly consistent with our hypothesis because the categorization of adjectives in relation to skin sensations required the same amount of time as the categorization of adjectives in relation to vision or hearing. The categorization of adjectives as unrelated to sensations (answers “no/not”) shows a greater number of significant differences; and categorization time in this case is increasing in the following direction: taste/olfaction, hearing, skin sensations, and vision. These results demonstrate that the categorization of adjectives as related or unrelated to a particular sense proceed in a very similar way.

### 2.3. The intensity of emotions reported during the mental reactivation of sense-related experience

**Fig 4:**
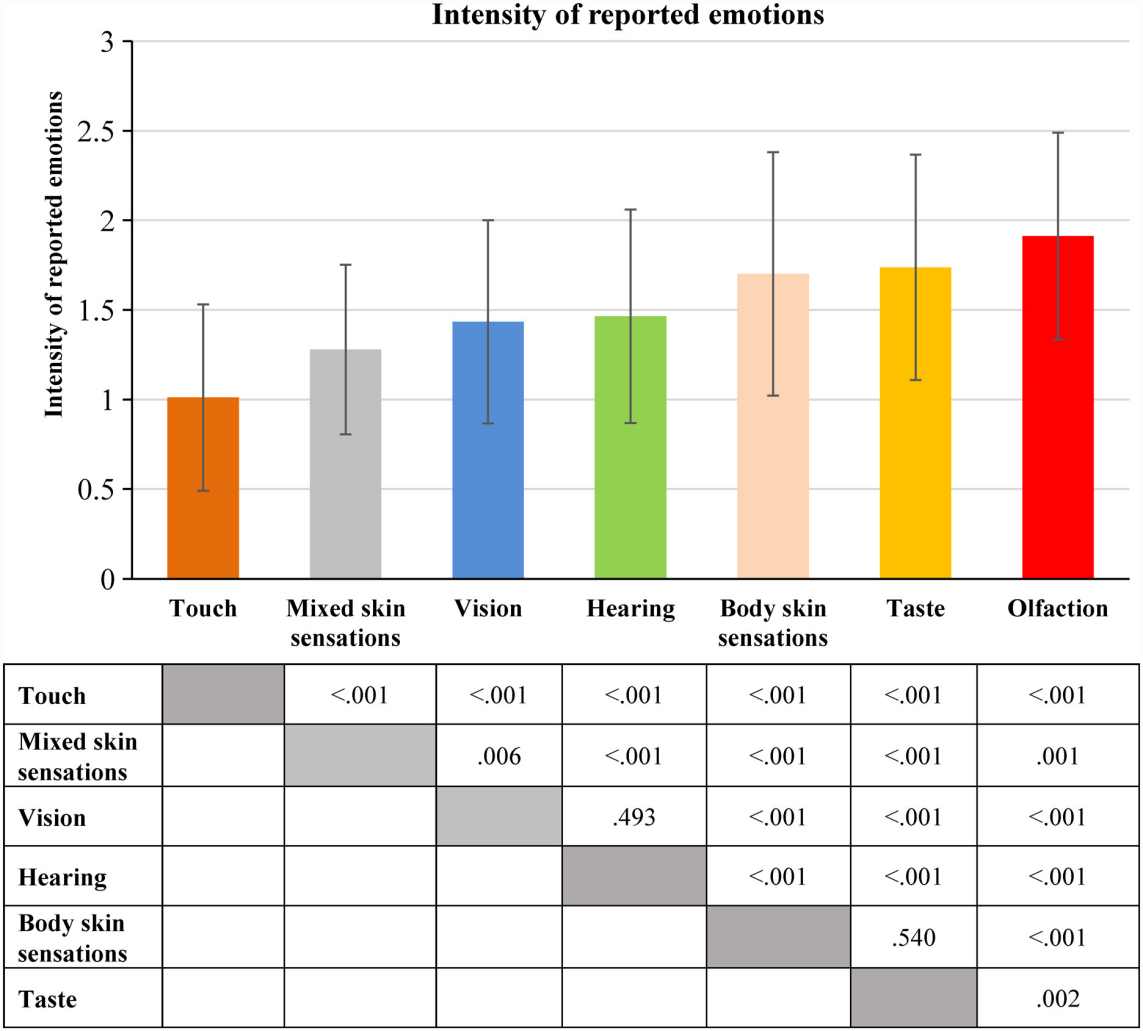
Intensity of emotions reported during the mental reactivation of sense-related experience (means and standard deviations). Median values of the intensity of reported emotions were calculated for each participant; this was done separately for each group of sense-related experience (see S2 Data). The distributions of the medians of the intensity of reported emotions were compared using Wilcoxon signed-rank test (N = 97). The statistical significance of the differences is displayed in the table under the figure.

We compared the absolute values of pleasantness ratings (the intensity of reported emotions) during the mental reactivation of experience predominantly related to different senses (Fig 4). Based on the results of the additional categorization of adjectives related to skin sensations (see the corresponding section in Materials), we analysed three types of experience related to skin sensations as experience predominantly related to (1) touch, (2) body skin sensations and (3) mixed skin sensations.

Results are in line with our hypothesis: the mental reactivation of experience predominantly related to olfaction, taste or body skin sensations was rated as causing more intense emotions than the mental reactivation of experience predominantly related to hearing, vision or touch. It should be noted that the mental reactivation of experience predominantly related to touch was rated as causing less intense emotions compared to the mental reactivation of experience predominantly related to all other senses.

**Fig 5:**
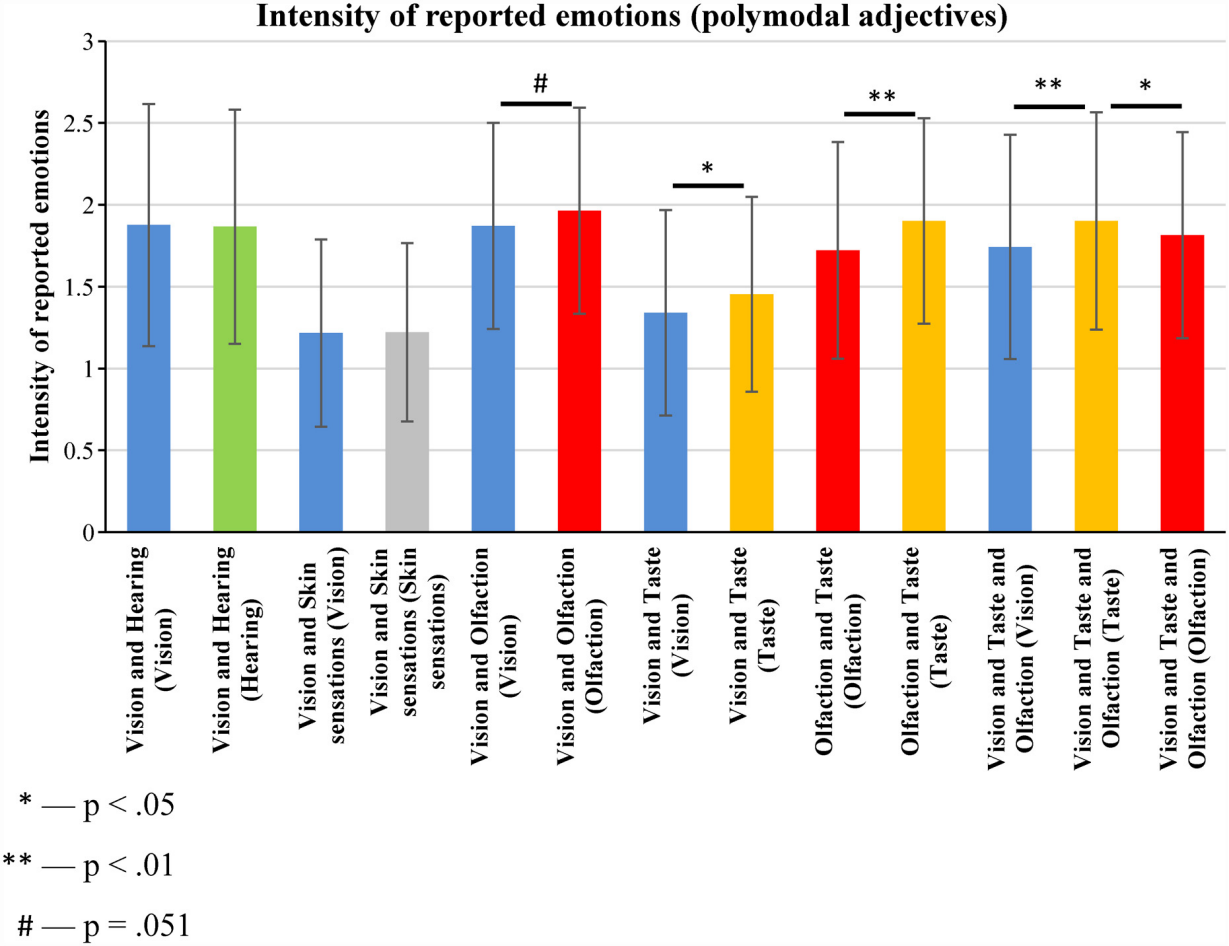
The intensity of reported emotions during pleasantness judgments of sense-related experience that was mentally reactivated using polymodal adjectives (means and standard deviations). The median values of the intensity of reported emotions were calculated for each participant; this was done separately for each type of sense-related experience (see S3 Data). The distributions of the medians of the time of the pleasantness judgment were compared using Wilcoxon signed-rank test (N = 97).

We also compared the intensity of emotions reported during pleasantness judgments of experience reactivated using polymodal adjectives (Fig 5). This means that the intensity of reported emotions was compared for experience that was mentally reactivated using the same adjectives but with different sense-related questions.

Fig 5 shows that food-related experience mentally reactivated by means of vision and taste-related adjectives or vision and taste and olfaction-related adjectives was rated as causing more intense emotions if it was reactivated using a taste-related question than if it was reactivated using a vision- or olfaction-related question.

### 2.4. The dynamics of skin resistance during the pleasantness judgment of mentally reactivated experience

**Fig 6:**
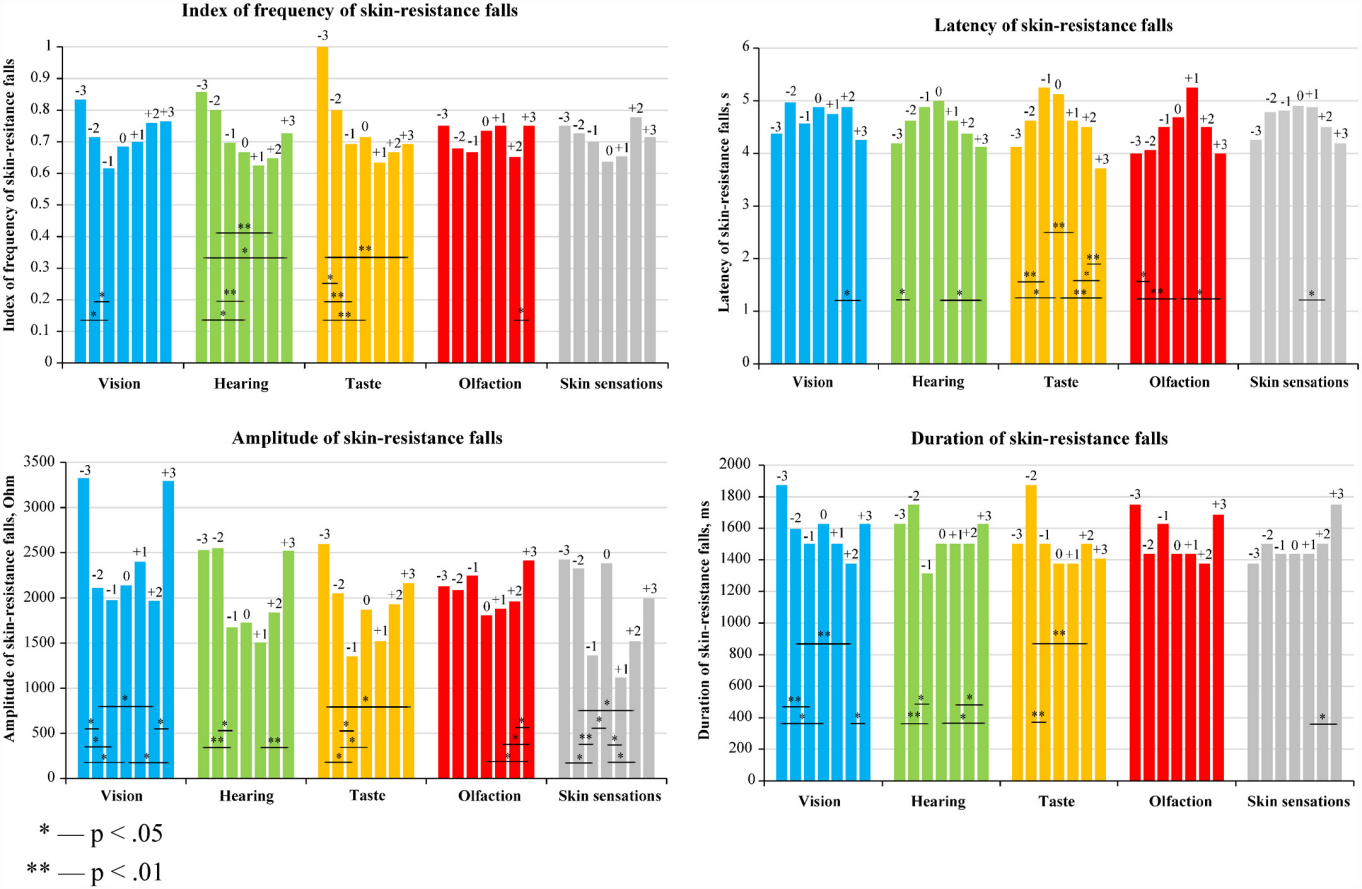
The characteristics of skin-resistance falls during pleasantness judgments of sense-related experience. Median values are shown separately for each of the ratings on a pleasantness scale. The distributions of the median values (or values in the case of index of frequency) were compared using Wilcoxon signed-rank test (N = 23).

Median values of the index of frequency, latency, amplitude and duration of skin-resistance falls (see S4 Data) were compared within the same sample of participants for those groups of trials when mentally reactivated experience had been rated differently on a pleasantness scale. (Fig 6).

The results shown in Fig 6 demonstrate 48 (of 240 comparisons: 12 comparisons x 5 senses x 4 characteristics of skin-resistance falls) statistically significant differences for experience that generated emotions varying in intensity within one valence. In line with our hypothesis, most of the revealed differences (45 out of 48; χ2 = 35.02; p < .01) can be described by the following relationship: a higher intensity of emotions was accompanied by a higher frequency, shorter latency, higher amplitude and longer duration of skin-resistance falls.

Fig 6 also demonstrates 9 (out of 60 comparisons: 3 comparisons x 5 senses x 4 characteristics of skin-resistance falls) statistically significant differences for experience that generated emotions that were equal in intensity but opposite in valence. They indicate that unpleasant emotions were more often accompanied by skin-resistance falls than pleasant emotions of the same intensity, and such skin-resistance falls had a higher amplitude, longer latency and duration in case of unpleasant emotions (except for olfaction-related experience). This result contradicts our hypothesis that the higher positivity of emotions reported during the mental reactivation of sense-related experience is accompanied by a higher frequency, shorter latency, higher amplitude and longer duration of skin-resistance falls.

### 2.5. Time of the pleasantness judgment of sense-related experience

**Fig 7:**
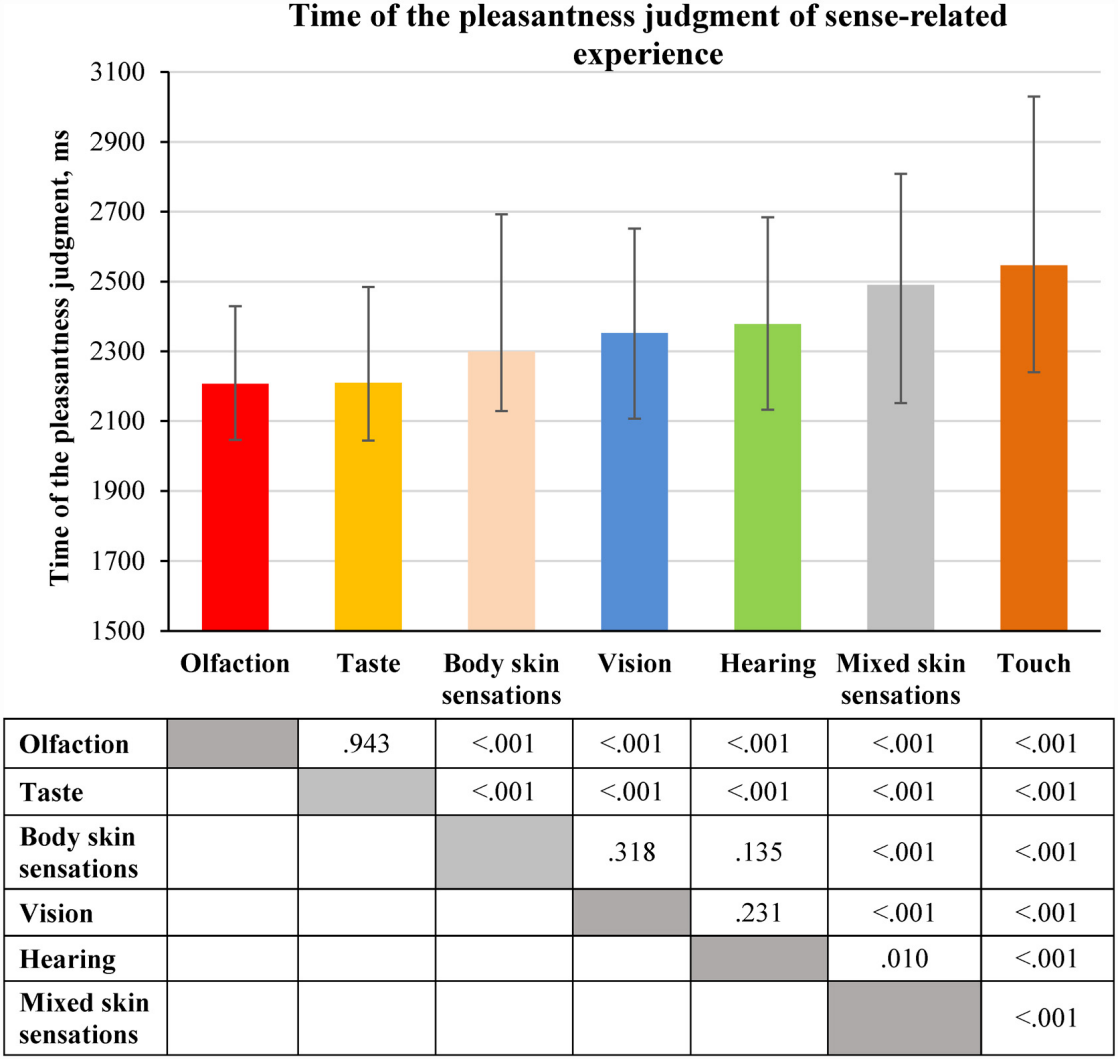
Time of the pleasantness judgment of sense-related experience (medians and interquartile ranges). The median values of time of the pleasantness judgment were calculated for each participant; this was done separately for each type of sense-related experience (see S5 Data). The distributions of the medians of the time of the pleasantness judgment were compared using Wilcoxon signed-rank test (N = 97). The statistical significance of the differences is displayed in the table under the figure.

By comparing the time of the pleasantness judgment of experience related to different senses (Fig 7) we found that it was longer for experience related to vision, hearing or various skin sensations than for that related to taste or olfaction (**the sense effect**).

This result is only partly consistent with our hypothesis because the pleasantness judgment of experience related to body skin sensations required the same amount of time as the pleasantness judgment of experience related to vision and hearing. However, the pleasantness judgment of experience predominantly related to touch took the longest time compared to the pleasantness judgment of experience predominantly related to all other senses.

**Fig 8:**
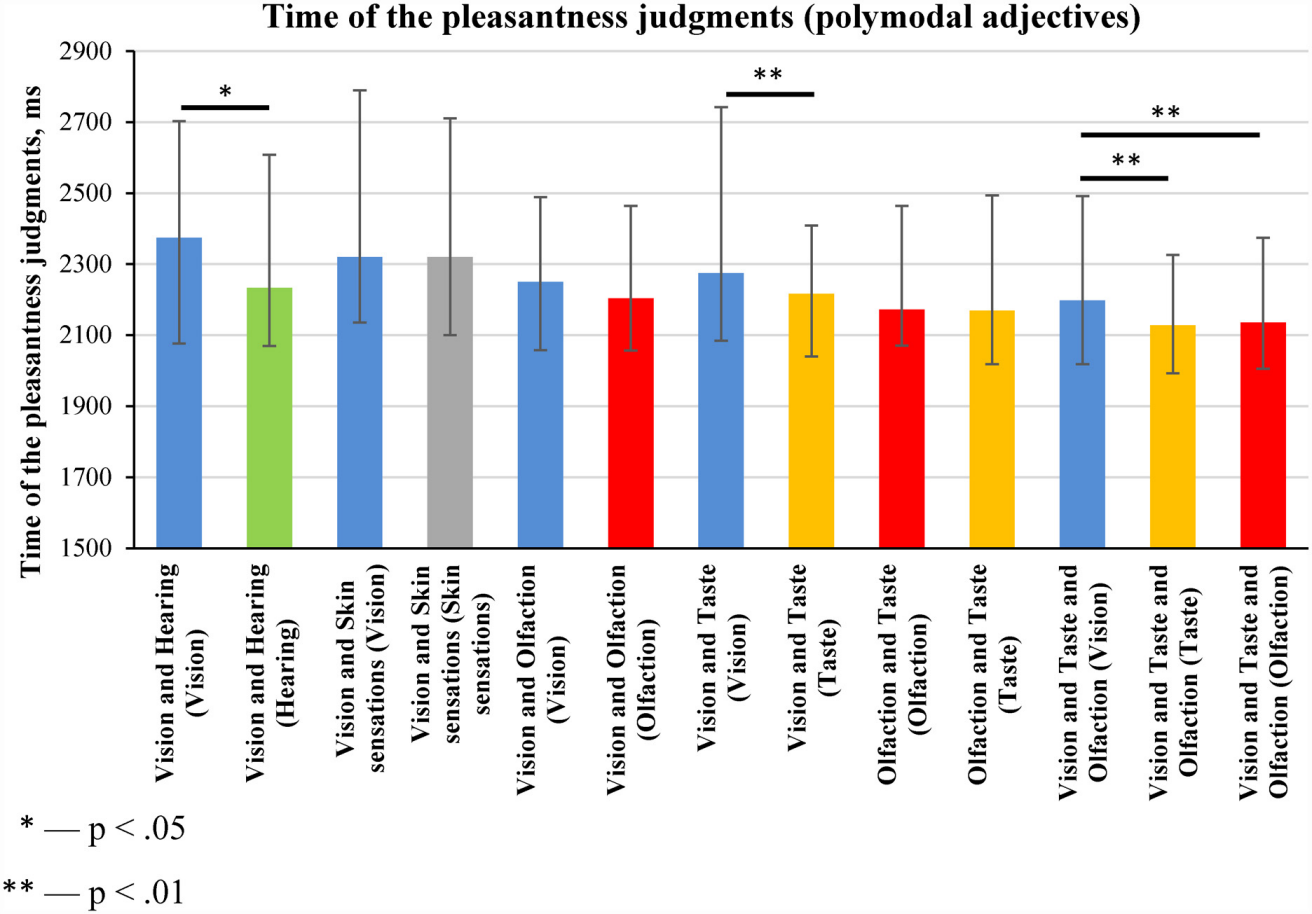
Time of the pleasantness judgments of sense-related experience mentally reactivated using polymodal adjectives (medians and interquartile ranges). The median values of the time of the pleasantness judgments were calculated for each participant; this was done separately for each type of sense-related experience (see S6 Data). The distributions of the medians of the time of the pleasantness judgments were compared using Wilcoxon signed-rank test (N = 97).

We also compared the time of the pleasantness judgment of experience reactivated using polymodal adjectives (Fig 8). This means that the time of the pleasantness judgements was compared for experience that was mentally reactivated using the same adjectives, but with different sense-related questions.

Fig 8 shows that the pleasantness judgments of vision and hearing-related experience took more time if an experience was reactivated using a vision-related question instead of a hearing-related question. The pleasantness judgments of food-related experience reactivated by means of vision and taste-related adjectives or vision and taste and olfaction-related adjectives took more time if experience was reactivated by means of vision-related question than by means of taste- or olfaction-related question.

The correspondence between the intensity of reported emotions, types of senses and time of the pleasantness judgment suggests that differences in the time of the pleasantness judgment are influenced by two factors: the specificity of a particular sense and the pleasantness level of the reported emotions. To differentiate the influences of these two factors, the medians of the normalized values of the time of the pleasantness judgment (Tukey normalization in SPSS 17.0) were compared using repeated-measures analysis with two within-subject factors for 5 senses (olfaction, taste, hearing, vision and skin sensitivity) × 7 pleasantness ratings (from -3 to +3). The analysis was conducted solely based on time data for 68 participants only; the reason for this choice is that there were missing values for 29 other participants who did not use all 7 ratings (from -3 to +3) for the pleasantness judgment of experience. This analysis demonstrated a significant effect of the sense type, F (4, 64) = 16.246, p < .0001, partial η^2^ = .504, and a significant effect of the pleasantness rating, F (6, 62) = 54.552, p < .0001, partial η^2^ = .841. The Sense × Pleasantness rating interaction was non-significant, F (24, 44) = 1.215, p = .281, partial η^2^ = .399.

**Fig 9:**
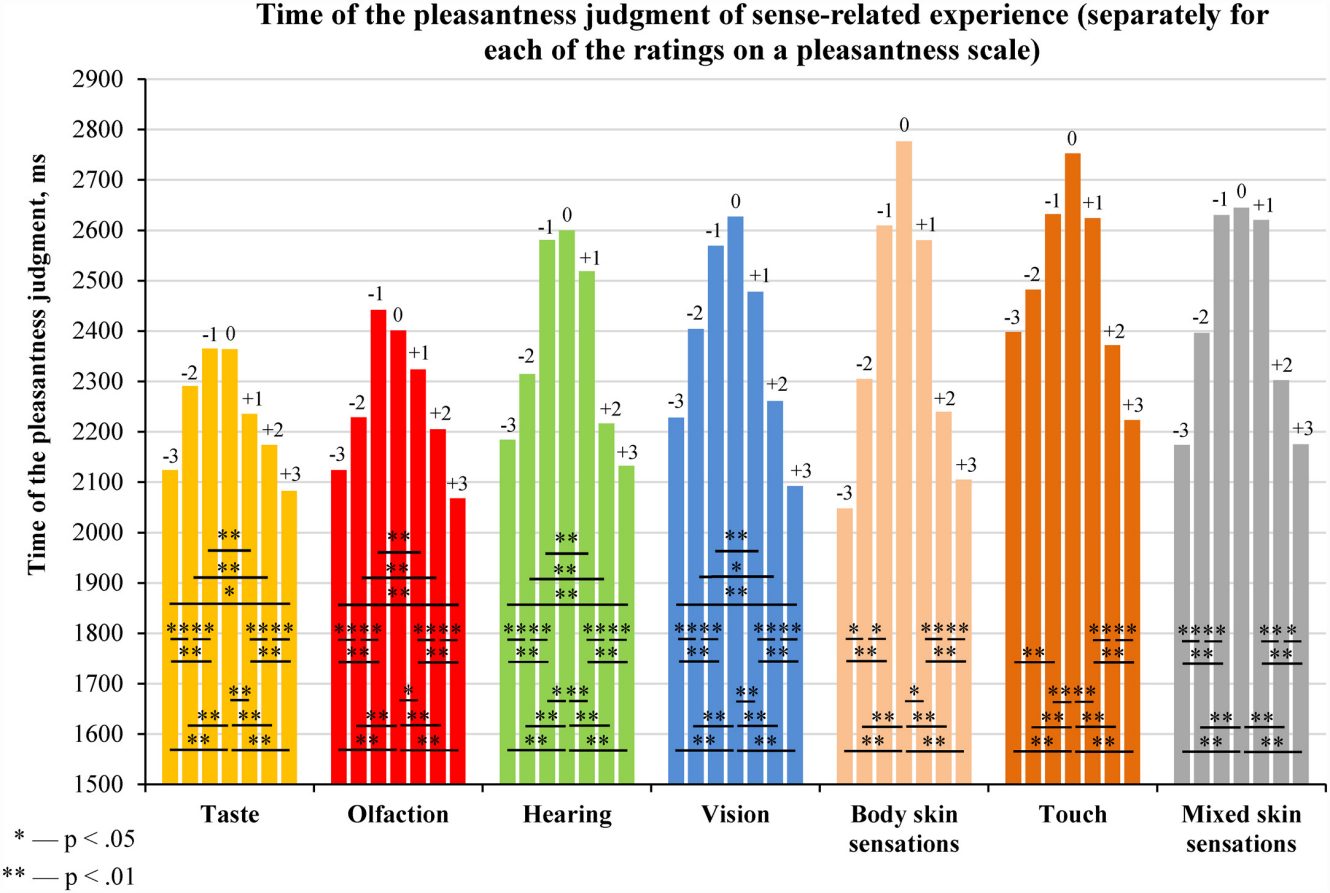
Time of the pleasantness judgment of sense-related experience, provided separately for each of the ratings on a pleasantness scale (medians). The median values of the time of the pleasantness judgment were calculated for each participant; this was done separately for groups of experience that were predominantly related to different senses (see S7 Data). The distributions of the medians of the time of the pleasantness judgment were compared using Wilcoxon signed-rank test (N = 97).

By comparing the time of the pleasantness judgment for each pleasantness rating (Fig 9) we found that it was longer for experience related to vision, hearing or various skin sensations than for that related to taste or olfaction (the above-described **sense effect**; the statistical significance of the differences is displayed in S1 Table).

Inverted U-shaped relations between the time of the pleasantness judgment and the intensity of the reported emotions observed for every sense (Fig 9) demonstrate the following **intensity effect**: the more intense emotions are reported (regardless of their positive or negative valence) during the pleasantness judgment of an experience, the less time the pleasantness judgment of that experience takes.

Comparisons of median time of the pleasantness judgment for opposite pleasantness ratings (Fig 9) revealed the following **valence effect**: when an experience generates negative emotions, the pleasantness judgment of that experience takes longer than for an experience causing positive emotions (but with equal intensity for both positive and negative emotions).

Both the intensity and the valence effects are in line with our hypothesis that the time of the pleasantness judgment negatively correlates with the intensity and pleasantness of the emotions reported during the mental reactivation of sense-related experience.

### 2.6. AoA and time of the pleasantness judgment of experience

We analysed AoA and age of experience acquisition (defined as predominantly related to early or late developed senses) as two factors that made equal contributions to the speed of the pleasantness judgment of experience. Medians of normalized time of the pleasantness judgment were computed separately for each participant for groups of experience that were predominantly related to different senses and reactivated using adjectives with different AoA. Medians of the normalized time of the pleasantness judgment were compared using repeated-measures analysis with two within-subject factors as follows: 5 senses (olfaction, taste, hearing, vision and skin sensations) × 4 AoA intervals (3–4 years, 5–6 years, 7–8 years and 9 years or later). The analysis revealed a significant effect of sense, F (4, 93) = 39.77, p < .0001, partial η^2^ = 0.631, and a significant effect of AoA, F (3, 94) = 95.32, p < .0001, partial η^2^ = 0.753. Sense × AoA interaction was also significant, F (12, 85) = 3.025, p = 0.01, partial η^2^ = 0.299.

**Fig 10:**
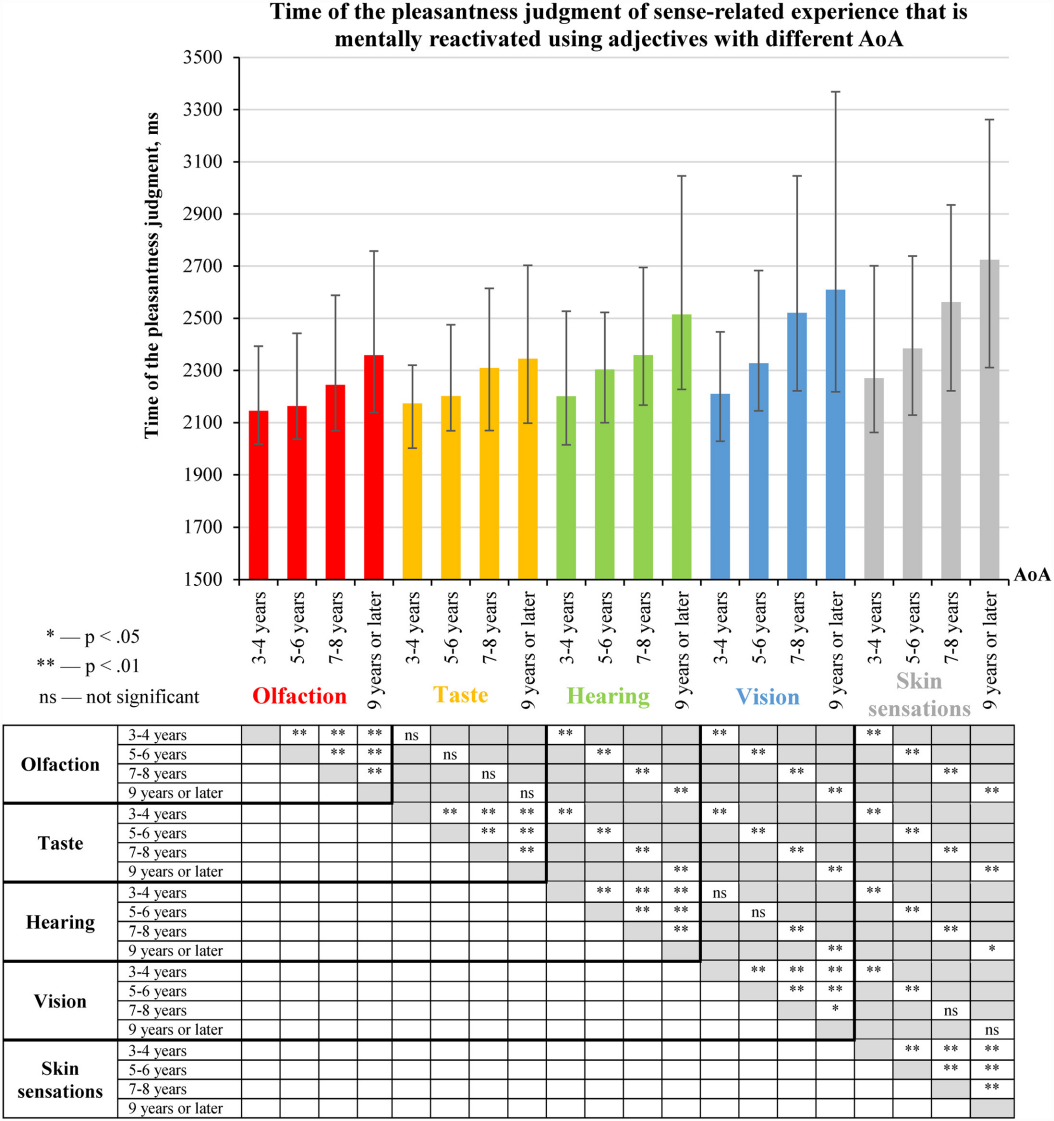
Time of the pleasantness judgment of sense-related experience that is mentally reactivated using adjectives with different AoA (medians and interquartile ranges). The median values of the time of the pleasantness judgment were calculated for each participant; this was done separately for groups of experience that were predominantly related to different senses and reactivated using adjectives with different AoA (see S8 Data). The distributions of the medians of time of the pleasantness judgment were compared using Wilcoxon signed-rank test (N = 97). The statistical significance of the differences is displayed in the table under the figure.

We also compared non-normalized time of the pleasantness judgment for groups of experience that were predominantly related to different senses and reactivated using adjectives with different AoA (Fig 10).

Fig 10 describes the **sense effect** (as previously illustrated in Fig 7) that is evident for groups of experience reactivated using adjectives with different AoA: pleasantness judgments of experience predominantly related to vision, hearing or skin sensations take more time than pleasantness judgments of experience predominantly related to taste or olfaction.

Fig 10 also shows the following **AoA effect**: the earlier the AoA of the adjectives, the less time was required for pleasantness judgments of experience that was mentally reactivated using these adjectives.

### 2.7. AoA and the intensity of reported emotions

**Fig 11:**
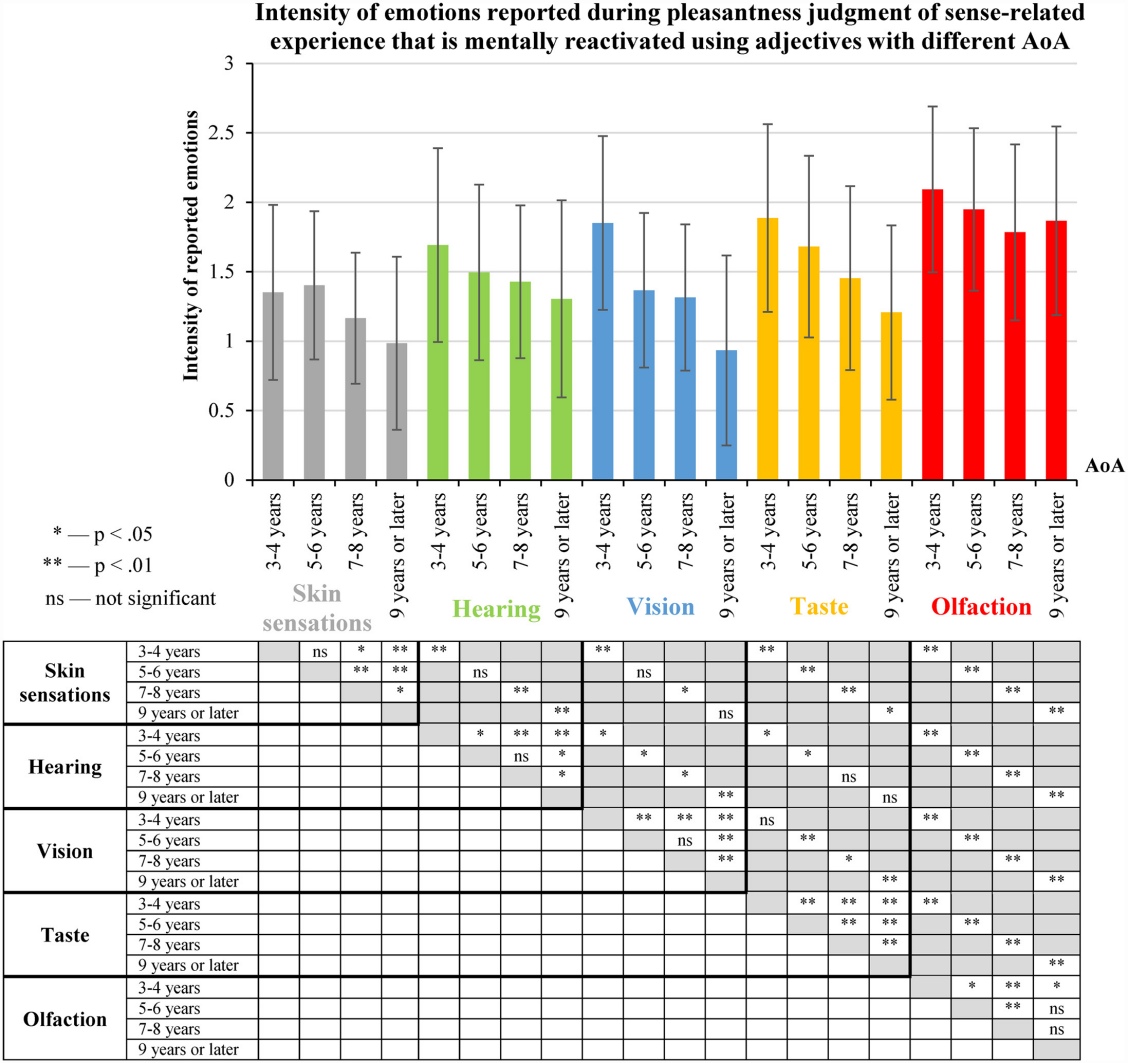
Intensity of emotions reported during pleasantness judgments of sense-related experience that is mentally reactivated using adjectives with different AoA (means and standard deviations). Median values of the intensity of the reported emotions were calculated for each participant for groups of experience predominantly related to different senses and reactivated using adjectives with different AoA (see S9 Data). The distributions of the medians of the intensity of reported emotions were compared using Wilcoxon signed-rank test (N = 97). The statistical significance of the differences is displayed in the table under the figure.

We compared the intensity of the reported emotions for groups of experience predominantly related to different senses and reactivated using adjectives with different AoA (Fig 11).

We found that the mental reactivation of experience predominantly related to olfaction or taste was rated as causing more intense emotions than the mental reactivation of experience predominantly related to hearing, vision or skin sensations (Fig 11).

Fig 11 also shows the tendency that the earlier the AoA of the adjectives, the more intense the emotions (regardless of their valence) reported by participants during the mental reactivation of experience by means of these adjectives. This result is consistent with our hypothesis.

### 2.8. AoA and pleasantness of reported emotions

We introduced a new Pollyanna index: the ratio of positive to negative pleasantness ratings (the quantity of positive ratings divided by the quantity of negative ratings, excluding zero ratings). The Pollyanna index was calculated for each group of sense-related experience rated by 97 participants of Experiment 2 (Table 3).

**Table 3:** The Pollyanna index for experience related to different senses and mentally reactivated by means of the adjectives with different AoA.

	Type of reactivated experience					
AoA of the adjectives	Vision	Hearing	Touch	Body skin sensations	Taste	Olfaction
**3–4 years**	6.69	2.02	2.58	2.65	4.75	3.60
**5–6 years**	2.15	1.48	1.15	3.77	2.82	1.60
**7–8 years**	1.25	1.21	2.79	4.98	1.63	0.91
**9 years or later**	4.43	1.87	1.99	5.58	1.39	0.45

As observed from Table 3, positive ratings prevail over negative ratings of experience related to all senses and reactivated by means of adjectives with all AoAs, except for late-acquired adjectives related to olfaction. Table 3 also shows that the pleasantness of reported emotions decreases, whereas the unpleasantness of reported emotions increases from early-acquired to late-acquired adjectives, except for adjectives related to touch and body skin sensations and late-acquired adjectives related to vision and hearing.

**Fig 12:**
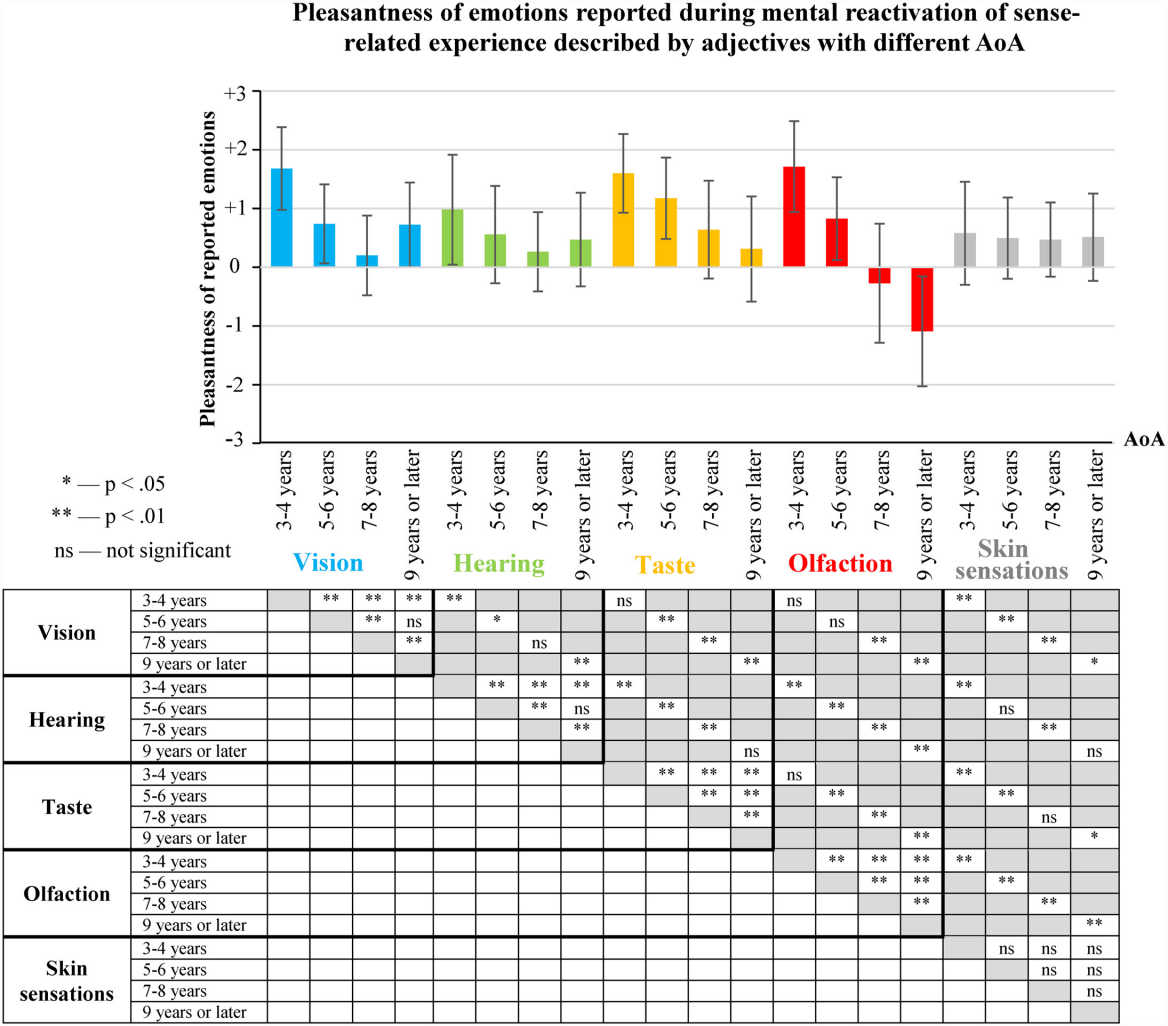
Pleasantness of the emotions reported during mental reactivation of sense-related experience described by adjectives with different AoA (means and standard deviations). Distributions of medians of the pleasantness of reported emotions were compared using Wilcoxon-rank test (N = 97). The statistical significance of the differences is displayed in the table under the figure.

To confirm this relationship, median values of the pleasantness of reported emotions were calculated for each participant for groups of experience that were predominantly related to different senses and reactivated by means of adjectives with different AoA (see S10 Data). Means and standard deviations of these distributions are shown in Fig 12.

### 2.9. Correlations between characteristics of sense-related adjectives and characteristics of the pleasantness judgment of sense-related experience

We correlated the imageability rating of every adjective with its frequency, length, number of syllables, median values for the intensity and pleasantness of emotions reported during the mental reactivation of experience, median values of the time of the pleasantness judgment and median values of AoA. Correlations were computed for every group of 120 adjectives related to each of five senses (Table 4).

**Table 4:** Spearman’s Rho correlations between the characteristics of adjectives related to different senses and characteristics of pleasantness judgments of experience mentally reactivated by means of these adjectives.

**Adjectives related to vision**							
	**2**.	**3**.	**4**.	**5**.	**6**.	**7**.	**8**.
**1. Intensity of reported emotions**	.57***	-.57***	-.28**	.21*	<-.01	.09	.10
**2. Pleasantness of reported emotions**		-.49***	-.37***	.15	.04	.15	.13
**3. Time of pleasantness judgment**			.56***	-.27**	-.18*	.14	.15
**4. AoA of the adjectives**				-.45***	-.37***	.20*	.18*
**5. Adjective frequency**					.33***	-.28**	-.32***
**6. Imageability of the adjectives**						-.15	-.20*
**7. Length**							.81***
**8. Number of syllables**							
**Adjectives related to hearing**							
	**2**.	**3**.	**4**.	**5**.	**6**.	**7**.	**8**.
**1. Intensity of reported emotions**	.29**	-.65***	-.16	.21*	.19*	-.07	-.10
**2. Pleasantness of reported emotions**		-.35***	-.02	.13	.21*	.05	.05
**3. Time of pleasantness judgment**			.45***	-.35***	-.55***	.39***	.39***
**4. AoA of the adjectives**				-.56***	-.54***	.42***	.39***
**5. Adjective frequency**					.29**	-.36***	-.35***
**6. Imageability of the adjectives**						-.31***	-.27**
**7. Length**							.91***
**8. Number of syllables**							
**Adjectives related to skin sensations**							
	**2**.	**3**.	**4**.	**5**.	**6**.	**7**.	**8**.
**1. Intensity of reported emotions**	.22*	-.54***	-.25**	.22*	.33***	-.18	-.12
**2. Pleasantness of reported emotions**		-.13	.00	.19*	.20*	.04	.10
**3. Time of pleasantness judgment**			.49***	-.55***	-.55***	.48***	.47***
**4. AoA of the adjectives**				-.51***	-.52***	.38***	.41***
**5. Adjective frequency**					.32***	-.46***	-.45***
**6. Imageability of the adjectives**						-.11	-.11
**7. Length**							.87***
**8. Number of syllables**							
**Adjectives related to taste**							
	**2**.	**3**.	**4**.	**5**.	**6**.	**7**.	**8**.
**1. Intensity of reported emotions**	.58***	-.54***	-.24**	.14	.33***	.12	.12
**2. Pleasantness of reported emotions**		-.57***	-.33***	.09	.47***	.08	.09
**3. Time of pleasantness judgment**			.41***	-.29**	-.49***	.18*	.16
**4. AoA of the adjectives**				-.47***	-.56***	.28**	.16
**5. Adjective frequency**					.15	-.52***	-.51***
**6. Imageability of the adjectives**						.02	.05
**7. Length**							.85***
**8. Number of syllables**							
**Adjectives related to olfaction**							
	**2**.	**3**.	**4**.	**5**.	**6**.	**7**.	**8**.
**1. Intensity of reported emotions**	-.02	-.54***	-.17	.16	.33***	.04	-.03
**2. Pleasantness of reported emotions**		-.25**	-.42***	.10	.19*	.14	.08
**3. Time of pleasantness judgment**			.49***	-.23*	-.52***	.11	.21*
**4. AoA of the adjectives**				-.56***	-.42***	.37***	.34***
**5. Adjective frequency**					.37***	-.48***	-.44***
**6. Imageability of the adjectives**						-.17	-.27**
**7. Length**							.87***
**8. Number of syllables**							

*—p < .05.

**—p < .01.

***—p < .001.

As observed from Table 4, the most of the correlation patterns are similar across adjectives. In particular, the time of the pleasantness judgments is negatively correlated with the intensity and pleasantness of emotions reported during the mental reactivation of experience; however, it is positively correlated with AoA for those adjectives used for mental reactivation. These data for sense-related groups of adjectives correspond to the above-described intensity effect, valence effect and AoA effect obtained for participants’ answers of different valence (positive and negative emotions). No significant correlation was observed between pleasantness scores and adjective frequency (except for adjectives related to skin sensations).

## Discussion

### Quantity of adjectives related to different senses

In this study, we found that vision-related adjectives predominate over adjectives describing all of the other types of sensations listed lexically in the Russian dictionary. These data are in line with previous results for other languages [18], [47]. High quantities of bimodal visual-haptic and olfactory-gustatory adjectives, along with the high modal exclusivity of hearing-related adjectives, are fully consistent with the data obtained with English adjectives [19–20]. Our results indicate that the quantity of adjectives related to the most differentiated experience based on the predominant use of vision exceeds the quantity of adjectives related to less differentiated experience based on the predominant use of hearing and early-developed senses, such as olfaction, taste and skin sensitivity.

However, a relatively small quantity of hearing-related adjectives was found in our study despite the fact that hearing relates to later-developed, highly differentiated experience. In our opinion, this can be discussed in relation to the fact that sounds are oscillatory motions and therefore, they are often described by means of verbs, not adjectives [74].

### Time of categorization

It was found that the categorization of adjectives in relation to vision, hearing or skin sensations required more time than the categorization of adjectives in relation to taste or olfaction. Connell and Lynott [75] obtained a similar pattern of results for English adjectives in a modality detection task. In this study, however, only the slowest categorization of adjectives was analysed in relation to the sense of touch (the tactile disadvantage effect). Connell and Lynott propose that the tactile disadvantage arises out of weak tactile endogenous control: people find it more difficult to sustain attentional focus on the tactile modality than on any other. Our data suggest another possible explanation. We believe that the time taken to categorize adjectives in relation to a particular sense reflects the number of functional systems predominantly related to this sense and therefore categorization time reflects differentiation of the structure of reactivated experience.

This suggestion is comparable with the data on the categorization of words: the more objects belong to a category, the more time is required to determine whether the word denotes an object from this category [76] (see, however, [77]). Similar results were obtained in studies of the priming effect [78]. Considering that experience related to early-developed senses (skin sensations, olfaction or taste) is less differentiated than experience related to later-developed senses (vision or hearing), the former should be mentally reactivated faster than the latter. However, our data showed that the categorization of adjectives in relation to skin sensations required more time in comparison with the categorization of adjectives in relation to other senses. It demonstrates that skin sensitivity, along with vision and hearing, predominantly relates to highly differentiated experience. This conclusion is consistent with our data on a large number of bimodal visual-tactile adjectives and data on the close relationship between language and manual gestures [79–81]. However, even in the early period of the development of experimental psychology, a similarity between the mechanisms of visual examination and touch was apparent, with some authors (Condillac, Sechenov, Wundt) asserting that hands are the teachers of eyes (for empirical evidence, see [82]). The contradiction can be eliminated if we assume that functional systems related to skin sensations form at different stages of ontogenesis and therefore, they may vary in the degree of differentiation. The verification of this assumption was one of the tasks performed in Experiment 2.

### Intensity of emotions reported during pleasantness judgments of experience related to different senses

This study has shown that the mental reactivation of experience predominantly related to olfaction, taste or sensations, perceived through the skin of the body, was rated as causing more intense emotions than the mental reactivation of experience predominantly related to hearing, vision or sensations, perceived through the tips of the fingers (touch). These results cannot be caused by any initial difference in the intensity of emotions predominantly related to different adjectives: selection of the adjectives for experiments was conducted solely on the bases of categorizing the relationship of one adjective to one or another type of sensation, with frequency and length of the adjectives being controlled.

Using an additional questionnaire [83] we revealed that olfaction and taste were dominant modalities of the imagination sphere in only 5 out of 96 participants of Experiment 2, thus excluding any significant interference by this factor on the obtained results.

It is unlikely that these results reflect only the connotative meanings highlighted by sensation-related adjectives, because reading or listening to sentences describing a behaviour causes an involuntary partial reactivation of the described experience ([11], [84–86]; among others).

**Fig 13:**
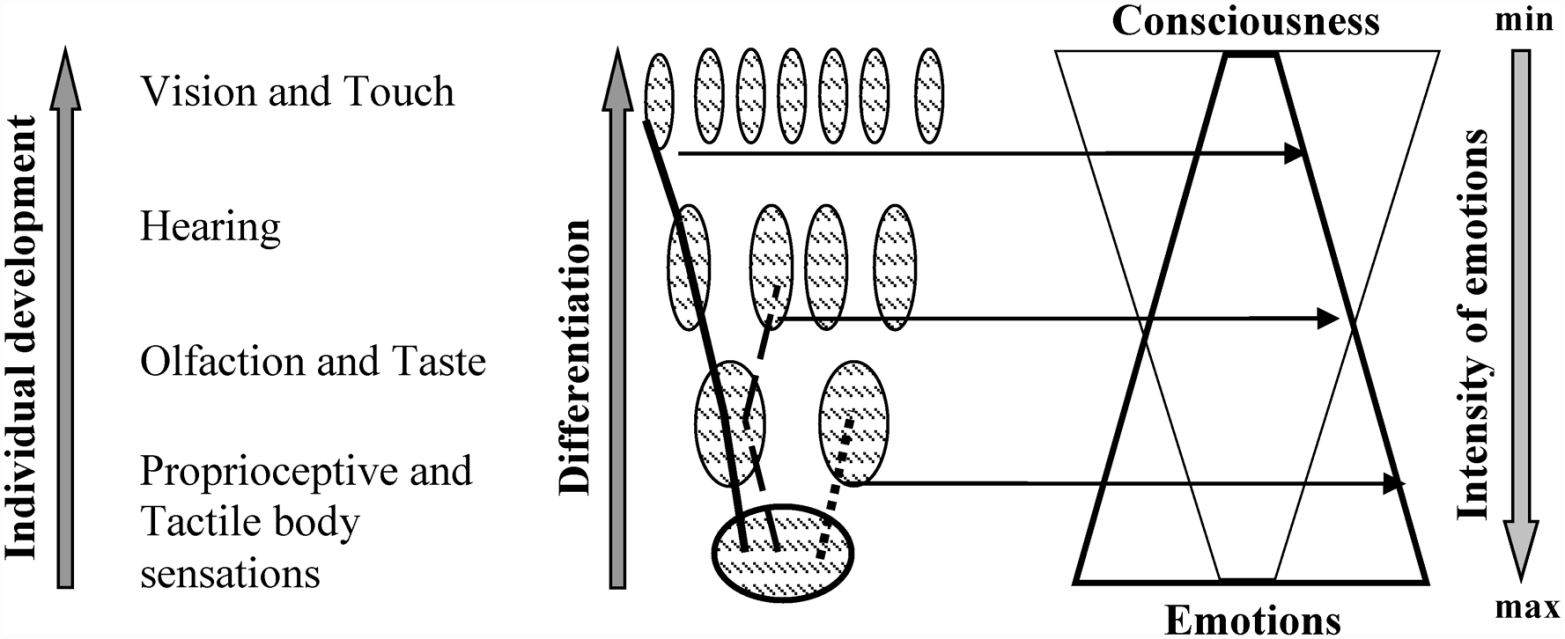
The sequence of formation of sense-related functional systems corresponded to different degrees of differentiation of experience and different intensity of emotions. The ovals depict functional systems formed at different stages of individual development. The ovals connected by the line illustrate the combination of functional systems that provide realization of experience. The trapeziums on the right side depict a decrease in the emotional characteristic and an increase in the consciousness characteristic of reactivated individual experience with increasing differentiation of experience. Slices at different levels of the trapezium depict the intensity of emotions generated during the realization of different sets of functional systems (ovals in the left part connected by a line).

In our opinion, the results of this study could be entirely explained based on the united concept of consciousness and emotions [55–58]. In this view, reactivation of early-formed, low-differentiated experience is more emotive than reactivation of later-formed, highly differentiated experience (see also [27], [37], [59–61], [63], [87–89]). Conceptual relationship between types of experience based on the activity of functional systems predominantly related to different senses, the degree of differentiation of these systems and intensity of emotions is shown in Fig 13.

As observed in Fig 13, the higher the proportion of “old” functional systems (whose activity underlies experience, i.e., systems formed at early stages of ontogenesis), the more pronounced is the emotional characteristic of the experience (intensity of emotions experienced by a subject) and the less pronounced is the consciousness characteristic of the experience. Therefore, we conclude that in the course of the mental reactivation of experience predominantly related to olfaction, taste or skin sensitivity of the body, a higher number of early-developed, low-differentiated functional systems becomes active within the structure of individual experience and participants rated their emotions as more intense than they did in the course of the mental reactivation of experience predominantly related to vision, hearing or touch.

Similar results were reported in the study of an emotional coefficient of different sensations [90]. Other empirical evidence shows that olfaction is predominantly promoted by the activity of low-differentiated functional systems: it was found that some olfactory bulb neurons in rabbits increase their activity with an increase in the preferability of the food offered [91] regardless of the specific type of food (see also [92]). Several studies have found that olfaction closely relates to emotions, but is much less closely related to words [93], [94]. Valence in the judgment of odours does not depend on the characteristics of odours but rather on an individual’s previous experience related to those odours [95], [96].

We believe that the high differentiation of experience predominantly related to vision, may be viewed as a result of the efficiency of visual simulation (imagination) for testing the possible ways of individual interaction with environment “in mind” instead of testing them in the environment, with the higher probability of the survival of organisms that have this “in mind” ability [97].

### The dynamics of skin resistance during pleasantness judgments of mentally reactivated experience

#### The intensity of reported emotions and skin resistance

In this study, the following relationship was found: the higher the intensity of pleasant or unpleasant emotions reported by participants during the mental reactivation of experience, the shorter the latency of skin-resistance falls; the higher the amplitude, the longer the duration of skin-resistance falls and the higher the frequency of skin-resistance falls.

These results are consistent with the data that the magnitude of the phasic component of skin conductance during the presentation of images with emotional content directly relates to the intensity (but not the valence) of experienced emotions rated by participants on a 7-point Likert scale [98]. Other studies have also found a significant positive correlation between subjective evaluations of IAPS images on the arousal scale and the magnitude of skin conductance during the presentation of those images [99–100]. Similar results were obtained for skin-conductance responses during the presentation of emotional and neutral sounds [101] and stimulating and relaxing musical excerpts [102]. It was also revealed that subjective evaluations of odours on an arousal scale directly related to the magnitude of skin conductance during the presentation of those odours [103]. Ratings on a scale measuring the intensity of experienced emotions were closely related to ratings on an arousal scale [100].

Skin-resistance falls have been found in situations of money loss [104]. It has also been found [105] that the presentation of negative words was accompanied by a significantly higher level of skin conductance than was the case regarding the presentation of neutral words. The magnitude of skin conductance during performance of the modified Stroop test in error trials was higher than in correct trials [106].

The common feature in the studies described above is that skin-resistance falls accompany intensification of the emotional characteristic of experience regardless of the negative or positive valence of emotions. This suggests that the presence of skin-resistance falls may be an indicator of an increase in the proportion of low-differentiated systems becoming active in the structure of individual experience.

#### Valence of reported emotions and skin resistance

A comparison of the characteristics of skin-resistance falls for experience rated as causing emotions of different valence but the same intensity reveals the following relationship: skin-resistance falls occurring during evaluations of experience as causing unpleasant emotions were more frequent and had a higher amplitude, longer latency and duration than skin-resistance falls occurring during the evaluation of experience as causing pleasant emotions. This effect is consistent with the data that skin conductance during implementation of the Stroop test is higher for words related to negative emotions than for words related to positive emotions [107]. Skin conductance is maximal when participants make facial expressions characterizing fear or disgust, weaker for anger and sadness, and minimal for happiness and surprise [108]. Similar results have been obtained in the above-mentioned study of relationships between odours, basic emotions and human life experience [95].

Significant positive correlations have been found between the amplitude and duration of the skin-resistance falls and the degree of the evaluated negativity of different tastes [109].

It can be concluded that our results are consistent with the data of other authors, but contradicting our hypothesis. Perhaps, the domain of negative experience (avoidance) has properties that distinguish it from the domain of positive experience (approach), but they are not reduced to a difference in the degree of differentiation of these domains.

### Time of the pleasantness judgment of experience

The **sense effect** found in this study can be explained by variations in the degree of differentiation of reactivated experience. Mental reactivation of highly differentiated experience related to vision, hearing or touch is associated with reactivation of a larger number of functional systems than mental reactivation of lower-differentiated experience related to taste and olfaction. The sense effect is consistent with the results obtained in Experiment 1 regarding the time required for categorizing adjectives as related to different senses.

The relationship between the domain reactivation time and the number of the elements forming this domain had been investigated before but explained based on other theoretical positions [110–112]. In our opinion, variation in the degree of differentiation of reactivated experience can be revealed when experience is characterized by varying degrees of awareness. It has been found that the amplitude of evoked potentials was higher for the conscious identification of signals than for the unconscious perception of signals [113]. The presentation of unmasked words is accompanied by activity in a greater number of brain regions and a greater number of neurons than presentations of masked words [114]. These data indicate that the activity of highly differentiated experience is based on a larger number of functional systems than is the activity of low-differentiated experience.

In our study, however, hearing-related adjectives were longer and included a greater number of syllables compared with all other sense-related adjectives. Many studies have shown that for 8-13-letter words the response time is greater for longer words than for shorter words (see, e.g., [115]). However, a positive correlation between the length of sense-related adjectives and AoA found in our and other studies [51], [116–117] may indicate that longer words are learned later in life. We believe that the high differentiation of hearing-related experience is manifested not only in the low intensity of emotions associated with such experience and the prolonged time of categorization and pleasantness judgments but also in the greater length of hearing-related adjectives.

The i**ntensity effect** found in this study corresponds to the data indicating that the evaluation of sounds, images and sentences as threatening required more time than the evaluation of sounds, images and sentences as neutral (non-threatening) ([118], see, however, [119]). Like our results, an inverted U-shaped pattern between response time and valence ratings of IAPS (International Affective Picture System) pictures is shown [120]. In this study, the inverted U-shaped pattern was also revealed between blood-oxygen-level dependence (BOLD) in the dorsal regions of the prefrontal cortex and valence ratings. These results indicate that the greatest activity in these brain regions was observed for neutral images, and it gradually decreased with the increase in the positivity and negativity of ratings. We assume that experience that is rated as causing intense emotions is low differentiated, i.e., it is based on the activity of fewer functional systems than highly differentiated experience, which is rated as causing low-intensity emotions. The fewer the functional systems that underlie an experience, the less time is required to reactivate the experience and evaluate it on a pleasantness scale.

The **valence effect** revealed in this study corresponds to the data that negative words typically elicit slower word naming and lexical decisions than neutral or positive words [121–122]. Similar results have been demonstrated with the Stroop test applied to adjectives denoting positive and negative personal qualities [123] and nouns denoting positive and negative emotions [107]. When sense-related adjectives (also used in our study) were presented as targets with the prime words “pleasant” and “unpleasant”, the categorization of targets as related to the prime took less time if “prime” and “target” were both positive than if they were negative [124]. However, negative words elicited faster valence judgments on a dichotomous positive-negative scale [125].

The asymmetry between positive and negative emotions is revealed in all spheres of the subject-environment interaction [123], [126]. In accordance with the united concept of consciousness and emotion [55–56], [58] and some other views [127–129], the domain of negatively rated experience (domain of avoidance behaviour) contains more functional systems; and these systems are more differentiated than those in the domain of positively rated experience (domain of approach behaviour). Negatively emotive words are predominant in the emotion lexicon [130]. It has been found that in trials in which the correct answer allowed participants to avoid losing money, the amplitude of N100 potential was greater than in trials in which participants received money as a reward for correct answers [131]. Those authors argue in favour of a direct relationship between the amplitude of N100 and the number of reactivated functional systems and consequently, the number of activated neurons specializing in those systems.

Our task was more complex than the task used in the study discussed above [125]: it did not imply the categorization of words into negative or positive, but instead required the partial reactivation of experience related to these words. This enabled us to reveal that experience within the domain of approach behaviour is less differentiated compared to experience within the domain of avoidance behaviour.

### AoA and pleasant judgments of experience

#### AoA and the time of the pleasantness judgment of experience

We found a long-standing **AoA effect** for experience related to all types of sensation which is consistent with other studies showing that words learned early in life can be recognised and produced faster than words learned later in life [51–54]. The AoA effect is usually explained by differences in representations of early- and late- acquired words (see, e.g., [51]). However, based on data demonstrating that the understanding of a word occurs only within relevant individual-environment interactions [1], [132–134], we assume that early-acquired adjectives are primarily associated with low-differentiated experience formed at early stages of development, whereas late-acquired adjectives are mainly associated with highly differentiated experience formed at later stages of development. In this view, the AoA effect is similar to the sense effect: the bases of these effects are associated with the assumption that a larger number of functional systems underlie the reactivation of highly differentiated experience compared to lower-differentiated experience.

Despite noticeable similarities between the sense effect and the AoA effect, it has been shown that the sense effect is independent of AoA because it was found among adjectives with different AoA. We suggest that experience predominantly related to different senses that people acquire in the course of their development has evolutional boundaries in differentiation and minimal thresholds for the complexity of individual experience, which enables the formation of such types of experience. Similar limitations probably restrict learning certain words at early stages of individual development.

#### Intensity of reported emotions and AoA

We showed that the earlier the AoA of adjectives, the more intense the emotions reported by participants during the mental reactivation of experience by means of these adjectives. The same relation between AoA and valence ratings is obtained for English abstract words [135]. High-intensity emotions induced by early-acquired words may be discussed in the context of the fact that the early stages of ontogenesis are considered as stages of integration of the phonological forms of words and sentences with information from the senses, autobiographical memory and emotions [136]. However, in accordance with the united concept of consciousness and emotion, a decrease in the intensity of reported emotions along with an increase in the AoA of adjectives can be explained by the general increase in the differentiation of experience implicit in these words. Thus, we have identified similar regularities for experience related to early- and later-acquired adjectives and experience predominantly related to early- and later-formed senses.

#### Pleasantness of reported emotions and AoA

It has been shown in this study that the earlier the AoA of the adjectives, the more positive the emotions reported by participants during the mental reactivation of experience by means of these adjectives. Such relation probably also reflects the general increase in the differentiation of experience in the course of individual development. However, the predominance of positive over negative ratings is evident for all types of experience, except for olfactory-related experience that is mentally reactivated by means of late-acquired adjectives. The “Pollyanna hypothesis” [137–138] is a possible explanation for these results. This bias towards positivity can help preserve health [139].

It is possible that experience predominantly related to olfaction includes more functional systems related to the negatively rated avoidance domain of experience than does experience predominantly related to other senses. The evolutionary significance of this mechanism may also well be explained in terms of health maintenance.

### Correlations of characteristics of sense-related adjectives and characteristics of mental reactivation of sense-related experience

We revealed a stable pattern of relationships that characterizes adjectives describing different sensations. The negative correlation between AoA and imageability has also been found for English words [116–117], [140], and Chinese hieroglyphs [141]. The negative correlation between the imageability of adjectives and the time needed for the pleasantness judgment corresponds to results obtained by other authors, who have shown that word imageability negatively correlates with word-naming latency of visually presented Chinese hieroglyphs [141] and response time during imageability rating [142].

We found a negative correlation between time of the pleasantness judgment and adjective frequency. This result corresponds to the data indicating that an increase of word frequency is accompanied by a reduction of response time [143–144]. The pleasantness of emotions reported by participants did not correlate with the frequency of the adjectives used for the mental reactivation of experience. This result does not correspond to the exposure effect [145].

### Conclusions

**Fig 14:**
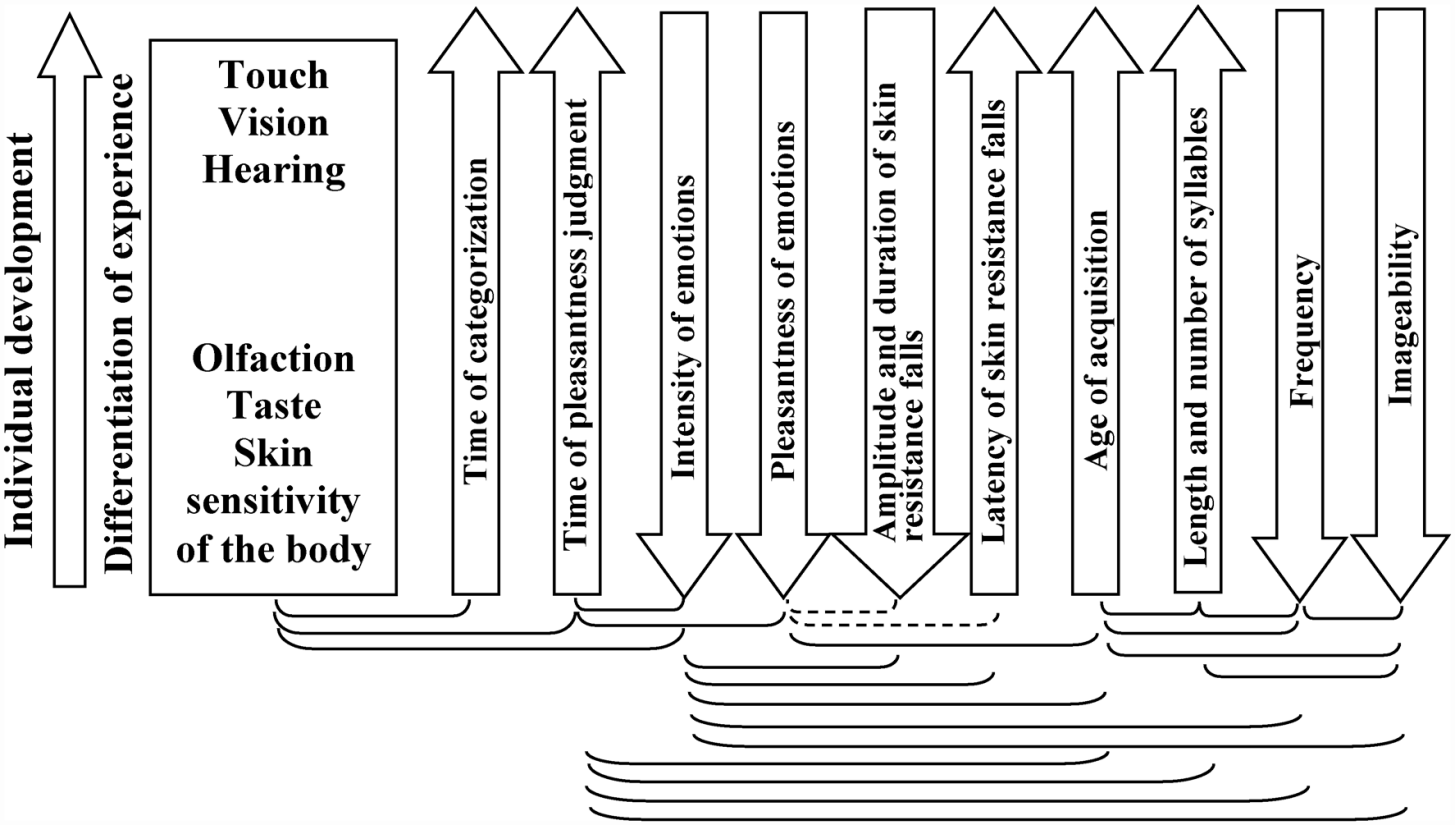
Correspondence between revealed and theoretically expected relationships between characteristics of mental reactivation of sense-related experience and characteristics of experience-related words.

The results of this study have been summarized in Fig 14. The arrows represent the theoretically expected relationships between various characteristics of the mental reactivation of sense-related experience and the characteristics of words relating to experience and used for its mental reactivation. The arrows indicate an increase in characteristics. The lines that connect the arrows indicate the relationships revealed in this study. The continuous lines connecting arrows indicate relationships that are consistent with our theoretical predictions, i.e., unidirectional arrows indicate a positive correlation between characteristics, and arrows running in the opposite direction show a negative correlation. The dashed lines connecting arrows indicate relationships that are inconsistent with our theoretical predictions, i.e., relationships that do not correspond to the direction of the depicted arrows.

Fig 14 shows that a high degree of differentiation of mentally reactivated experience corresponds to the decreased intensity and pleasantness of emotions, along with the need for prolonged time to make the pleasantness judgment, decreased imageability and frequency and increased AoA and length of the adjectives used for the mental reactivation of this experience. Only the characteristics of skin-resistance falls as they relate to the pleasantness of emotions reported by participants during the mental reactivation of sense-related experience fail to correspond to the theoretical predictions. However, this finding is consistent with the data of other authors (see Discussion). The results of this study correspond to the theoretical proposition that the sequence of the ontogenetic formation of experience predominantly related to different senses is an indicator of differences in the degree of differentiation of that experience. Experience that is predominantly related to olfaction, taste or skin sensitivity of the body is low differentiated, whereas experience that is predominantly related to vision, hearing or touch is highly differentiated. This is reflected in the characteristics of the mental reactivation of experience: the speed of the categorization and pleasantness judgment corresponds not only to the intensity and valence of the emotions reported during the mental reactivation of experience (as described from the first-person perspective) but also to the AoA, frequency, length and imageability of adjectives related to the reactivated experience.

## Supporting Information

S1 Appendix Sense-related adjectives used for the mental reactivation of sense-related experience (Experiment 2). (XLS)

S2 Appendix Detailed description of the method. (DOC)

S1 Code The source code archive of the experiment software (Experiments 1 and 2). (ZIP)

S1 Data Categorization time. (XLS)

S2 Data Intensity of reported emotions. (XLS)

S3 Data Polymodal adjectives: intensity of reported emotions. (XLS)

S4 Data Skin resistance data. (XLS)

S5 Data Time of the pleasantness judgment. (XLS)

S6 Data Polymodal adjectives: time of the pleasantness judgment. (XLS)

S7 Data Time of the pleasantness judgment (separately for each of the ratings on a pleasantness scale). (XLS)

S8 Data Time of the pleasantness judgment (separately for groups of sense-related experience that were reactivated using adjectives with different AoA). (XLS)

S9 Data Intensity of reported emotions (separately for groups of sense-related experience that were reactivated using adjectives with different AoA). (XLS)

S10 Data Valence of reported emotions (separately for groups of sense-related experience that were reactivated using adjectives with different AoA). (XLS)

S1 Table Statistical significance of the differences in the median values of the time of the pleasantness judgment of experience predominantly related to different senses (Sense effect). (DOC)
