# Supplementary material for: Mental Reactivation and Pleasantness Judgment of Experience Related to Vision, Hearing, Skin Sensations, Taste and Olfaction
Source: PLoS One. 2016 Jul 11;11(7):e0159036. doi: 10.1371/journal.pone.0159036 (PMC4939968; doi:10.1371/journal.pone.0159036)
Supplement: S2 Appendix — (DOC) [file pone.0159036.s002.doc]

**Material and Methods**

**The initial categorization of adjectives**

The initial categorization of adjectives was done by 44 native Russian-speaking psychology students (23 women, aged 17 to 28, median age 18). All of the adjectives (15,918 in total) listed in the Russian dictionary [71] were printed in random order on 94 worksheets. Participants received these worksheets with adjectives and a sheet with instructions in Russian: “Просим Вас поставить крестик в клеточке рядом с теми прилагательными, которые не относятся ни к одному из ощущений, получаемых с помощью глаз, ушей, носа, языка, рук, тела, а также не относятся к ощущениям внутренних органов. Не задумывайтесь над ответом, доверяйте своему первому впечатлению. Если прилагательное Вам совсем не знакомо, поставьте в клеточку рядом с ним букву «н»”. The English translation of the instruction was as follows: “Please mark with a cross the box next to those adjectives that do not describe any of sensations received through eyes, ears, nose, tongue, hands, body, and sensations of internal organs. Do not dwell on the answer: trust your first impression. If the adjective is not familiar to you, mark it with the letter ‘n’ in the box next to it”. The participants performed the task at their leisure over a month.

Using binomial m criteria, we tested whether the frequency of answers “not related” and “not familiar” for each adjective was greater than 50% probability. The results showed that 8,302 adjectives were classified by 31 or more participants as either unrelated to senses or unknown (p < .01) and were excluded from further research. The remaining 7,616 adjectives were used for computerized categorization.

**Experiment 1. Сomputerized сategorization of adjectives into different senses**

**Subjects.** Computerized categorization of adjectives was done by a sample of 115 native Russian-speaking psychology students (79 women, aged 17 to 36, median age 19) and by an additional sample of 13 students (10 women, aged from 17 to 23, median age 18). Each participant received payment (the equivalent of $5 in roubles).

**Materials.** The adjectives (7,616 overall) selected in the course of the initial categorization were randomly divided into three lists of 2,538, 2,539 and 2,539 adjectives. Each participant rated only one list of adjectives during the computerized categorization. An additional sample of 13 participants received all three lists of adjectives and performed the task in several sessions (one session per day).

**Procedure.** Each participant was seated in a quiet testing room approximately 50 cm from the computer display. A standard white keyboard for Windows (qwerty) was used, with the “G” and “H” keys labelled as “нет” and “да” (Russian equivalent of “no/not” and “yes”, Times New Roman, 16, bold). A separate group of 36 female participants performed the task with the keys for “no/not” and “yes” placed in the reverse order. The “F” and “J” keys were removed to prevent participants from pressing the wrong keys. The central part of the space bar was covered with 3 cm of blue tape. The other keys were covered with white, non-transparent sticky tape.

We used a custom experiment software. The software was written for Microsoft Windows XP in C++ with the use of DirectX. DirectDraw was used for accelerated graphics rendering in full-screen mode. DirectInput was used to achieve minimum key-press time measurement delays and deviation. The source code archive of the experiment software is attached in the supplementary materials (S1 Code).

At the beginning of the study, every participant had two 1-minute training sessions with 10 presentations of the word “yes” and 10 presentations of the word “no/not” in random order. In the course of the training sessions, participants learnt to use the keyboard in a specific manner (see below), and we obtained timing data about decision making for every response key.

Prior to the experimental sessions, the participants read printed instructions. The main study consisted of five blocks. Each block contained five sessions. In the course of each session, one of the sense-related sentences was permanently presented in the middle of the screen in the white frame against the black background (Table 2).

**Table 2. Sense-related sentences for computerized categorization of adjectives.**

| **Sense-related sentences** | **English translation** |
| --- | --- |
| Эту характеристику объекта я оцениваю зрительно. | I perceive this characteristic of an object by vision. |
| Эту характеристику объекта я оцениваю на слух. | I perceive this characteristic of an object by hearing. |
| Эту характеристику объекта я оцениваю по запаху. | I perceive this characteristic of an object by smell. |
| Эту характеристику объекта я оцениваю на вкус. | I perceive this characteristic of an object by taste. |
| Эту характеристику объекта я оцениваю на ощупь. | I perceive this characteristic of an object by touch. |

Participants were instructed to look at the screen keeping the space bar pressed using the index finger of the dominant hand. One of the adjective appeared for 1000 ms underneath the sense-related sentence. Participants were asked to press the key labelled “yes” if they agreed or “no/not” if they disagreed that the presented adjective describes what that can be perceived in the sensory modality featured in the sense-related sentence. Participants were asked to wait for an adjective to disappear before giving an answer. To give an answer, participants had to stop pressing the space bar and press either the key labelled “yes” or the key labelled “no/not”; they were then required to return their index fingers to the central (coloured) part of the space bar. The next adjective appeared 700 ms after participants resumed pressing the space bar. This procedure allowed us to measure the time required to categorize each adjective: the interval between appearance of an adjective on the screen and releasing the space bar.

In the course of one session, 104 or 105 adjectives were successively presented underneath one of the sense-related sentences (the first three presented adjectives were considered as training). Next, the sentence was changed and a new session was started. All five sense-related sentences were presented only once in each block. Participants took a rest break after each block. The experiment lasted 3-4 hours.

The order of the first three training adjectives was the same for all participants. The experimental adjectives (2,538 or 2,539 adjectives) were randomly divided into 25 session lists. The adjectives within each session list were presented in a pseudorandom order allowing a random presentation of the entire set without repetition. The combination of the session lists of adjectives and sense-related sentences was counterbalanced across subjects (for example, one session list of adjectives was presented under a vision-related sentence for one participant and under a hearing-related sentence for another participant, etc.). Consequently, every adjective was assessed by 9-13 participants underneath each sense-related sentence.

**Experiment 2. Pleasantness judgment of experience mentally reactivated by means of sense-related adjectives**

**Subjects.** An independent sample of 97 native Russian-speaking psychology students (70 women, aged 15 to 26, median age 18) assessed pleasantness of experience as mentally reactivated through sense-related adjectives. There was only one 15-year-old participant (university student) and no 16-year-old participants in the study. Each participant received payment (the equivalent of $5 in roubles). Skin resistance was measured in 23 out of 97 participants (14 women, aged 17 to 25, median age 18).

**Materials.** Based on the results of Experiment 1, we selected 475 adjectives (data are available in the S1 Appendix) with equalized frequency, length and number of syllables. During this selection, we aimed to include the maximum possible number of different multimodal adjectives. These 475 adjectives included 34 vision-related, 111 hearing-related, 71 skin sensitivity-related, 61 taste-related, 64 olfaction-related, 9 vision and hearing-related, 49 vision and skin sensitivity-related, 21 vision and taste-related, 18 vision and olfaction-related, 20 taste and olfaction-related, and 18 vision and taste and olfaction-related adjectives. The resulting lists contained 120 adjectives related to one of the senses, i.e., vision, hearing, skin sensitivity, olfaction and taste (properties of these sense-related adjectives comprising each list are presented in Table 1). We also introduced 25 additional adjectives (5 adjectives for each of the senses) to use in training before each session.

**Procedure.** Participants were seated in a quiet testing room approximately 50 cm from a computer display and keyboard. The keys “D”, “F”, “G”, “H”, “J”, “K”, and “L” were labelled, respectively, as “-3”, “-2”, “-1”, “0”, “+1”, “+2”, and “+3” (Times New Roman, 18). The central part of the space bar was covered with 3 cm of blue tape. Other keys were covered with white, non-transparent sticky tape. We used the same custom software as in Experiment 1.

Each participant was given a 5-minute training session at the beginning and after the third and the fifth blocks of the study. Each of the numbers “-3”, “-2”, “-1”, “0”, “+1”, “+2”, “+3” was presented 10 times for 500 ms in random order during the training session. In the course of the training sessions, participants learnt to use the keyboard in a specific manner (see below), and we received individual timing data about simple decision making for every response key.

Prior to the experimental sessions, each participant received oral instructions from a researcher and read a brief instruction in Russian on the computer screen “Вам предлагается оценить, насколько приятны или неприятны ощущения, возникающие у Вас в различных ситуациях. Для этого используйте 7-балльную шкалу от +3 до -3, где +3 — очень приятные, -3 — очень неприятные, 0 — нейтральные. При оценке старайтесь сосредоточиться именно на возникающих у Вас ощущениях. Нажмите и удерживайте клавишу ПРОБЕЛ”. The English translation of the instruction was as follows: “You are required to assess how pleasant or unpleasant your feelings are in different situations. Use a 7-point scale from +3 to -3 for assessment with *very pleasant* at +3, *very unpleasant* at -3 and *neutral* at 0. Try to focus only on your feelings when assessing. Press and hold the space bar”.

The main study consisted of five blocks. Each block contained five sessions. In the course of each session, one incomplete sense-related question (white letters against black background) was permanently presented in a frame in the middle of the screen (Table 3).

**Table 3. Sense-related questions for the mental reactivation of experience.**

| **Sense-related questions** | **English translation** |
| --- | --- |
| Что вы переживаете, когда видите объект | What do you feel when you see an object |
| Что вы переживаете, когда слышите звук | What do you feel when you hear a sound |
| Что вы переживаете, когда чувствуете запах | What do you feel when you sense a smell |
| Что вы переживаете, когда ощущаете вкус | What do you feel when you sense a taste |
| Что вы переживаете, когда трогаете объект | What do you feel when you touch an object |

Participants were instructed to look at the screen while keeping the space bar pressed using the index finger of the dominant hand. One appropriate adjective appeared for 1500 ms underneath the sense-related question. Participants were asked to wait for the appearance of an adjective and then to try to imagine the behaviour described in a complete question (e.g., “What do you feel when you sense a taste *bitter*”). Next, the participants had to assess the un/pleasantness of generated emotions by pressing one of the seven response keys on the keyboard, with very pleasant at +3, very unpleasant at -3 and neutral at 0. Participants were not time-limited when assessing their emotions but they were advised not to spend much time thinking about their response because the “first feelings” are usually the most reliable and of prime interest in this study. The participants were asked to wait for the adjective to disappear before giving an answer.

To give an answer, participants had to stop pressing the space bar, press the corresponding response key with their index finger, and then resume pressing the central part of the space bar. The next adjective appeared 5 seconds after participants resumed pressing the space bar. This technique allowed use to measure the time required for the pleasantness judgment of each adjective, i.e., we were able to measure the interval between the appearance of an adjective and the release of the space bar. In the course of the session, 25 appropriate adjectives were successively presented (the first adjective was training). Next, the question was changed and a new session started. All five questions were presented once for each block.

Experimental adjectives were presented in random order without recurrence underneath the corresponding sense-related questions. The presentation order was independently randomized for each participant. Multisensory adjectives were presented once under each corresponding question. The order of training adjectives was the same for all participants. The order of presentation of five questions through five blocks was counterbalanced in a Latin square format. Thus, over the course of the study 625 adjectives (120 adjectives for each sense and 25 training adjectives) were presented. Participants took a rest break after each block. The experiment lasted 3-4 hours.

**Analysis of response time in the course of training sessions.** Differences in the time of the pleasantness judgments obtained in the course of experimental sessions can be accounted for by differences in the position of response keys: the keys labeled “+3”, “+2” and “+1” were located to the right from the keys labeled “-3”, “-2” and “-1”; and the keys labeled “+3” and “-3” were located more laterally than the keys labeled “+2” and “-2”. To test this hypothesis we compared median values of response time for each of the response keys in the course of training sessions (see S7 Data) using Wilcoxon signed-rank test (N = 97). It was found that response time for the keys “-3” and “+3” differed in the training sessions in the same way as in the experimental sessions (p < .001). Response time for the keys “-1” and “+1” differed in the training sessions in the opposite direction compared to the experimental sessions (p = .02). There was no difference in response time between the keys “+2” and “-2”. Keys “+3”, “+2” and “+1” in the training sessions differed in the same way as in the experimental sessions (p < .01). However, there was no difference in response time for the keys “-3” and “-2”, or “-3” and “-1”. Other seven comparisons of response times for different response keys in the training sessions showed significant differences (p < .05) but in the opposite direction to those obtained in the experimental sessions.

**AoA of the adjectives related to different senses**

An independent sample of 107 native Russian-speaking students (87 women, aged 18 to 32, median age 19) reported the AoA of 475 adjectives relating to different senses (vision, hearing, skin sensitivity, olfaction and taste) as used in Experiment 2 for the mental reactivation of experience (see Table 1). To define the AoA of adjectives, we used a modified method of subjective rating of AoA [73].

Adjectives, regardless of their relationship to senses, were printed in random order in two columns on a single page. Seven choices were given to the right of each adjective as follows, 0-2, 3-4, 5-6, 7-8, 9-10, 11-12, 13…. Participants received one page of adjectives and were asked to read the instruction at the top of the page “Пожалуйста, укажите (обведя кружком) возраст, в котором вы узнали слова, написанные на этом листе. Если вы не знаете значение слова, поставьте крестик рядом с ним. Возраст, в котором вы узнали слово, означает возраст, в котором вы начали понимать слово, когда кто-то говорил его вам, ДАЖE ЕСЛИ в этом возрасте ВЫ НЕ МОГЛИ говорить, читать, или писать это слово”. The English translation of the instruction was as follows: “Please circle the age range at which you learned each of the words on the page. An approximate age is good enough for this rating. If you do not know the meaning of a word, just write an X near the word. By ‘learning a word’ we mean the age at which you would have understood that word if somebody had used it in front of you, EVEN IF YOU COULD NOT use, read or write it at the time”. No time limit was placed on responses. The experiment lasted 15-20 minutes. Participants received one page of adjectives per week (five pages in total).

Not all of the participants rated all five lists of the adjectives. Every adjective was presented to 76-85 participants. Responses were placed on an 8-point scale (from 1 for age 0 to 2, to 7 for age 13 or older, and 8 for unknown adjectives). The median value of AoA was computed for every adjective. Based on the median values of AoA, we calculated the percentage of adjectives with different AoA among adjectives related to different senses (see Table 1). Because of the very small number of adjectives with AoA in the range of 11 years and older, those adjectives were pooled with the adjectives with AoA in the range between 9 and 10 years in all computations.

**Imageability of sense-related adjectives**

An independent sample of 60 native Russian-speaking students (52 women, aged 17 to 33, median age 18) rated the imageability of 475 adjectives related to various senses (vision, hearing, skin sensitivity, olfaction and taste) as used in Experiment 2 (see Table 1). Because some adjectives described more than one type of sensation, they were presented more than once, extending the total number of word presentations to 600.

The adjectives comprised 5 worksheets containing 2 columns of 120 randomly printed adjectives, relating to one of the senses: vision, hearing, skin sensitivity, olfaction and taste. One of the following words was printed to the right of each adjective: “объект” (“object”) for adjectives describing visual or skin sensations, “звук” (“sound”) for adjectives describing auditory sensations, “вкус” (“taste”) for adjectives describing taste sensations, and “запах” (“smell”) for adjectives describing olfactory sensations. There was an empty box to the right of each word combination. A question was printed at the top of each worksheet. For adjectives related to vision, the question was: “Can I imagine what I see?” (translated into English, Russian original “Могу ли я представить, что вижу ?”). For adjectives describing auditory, tactile, taste and olfactory sensations, the verb “see” was changed to “hear” (“слышу”), “touch” (“трогаю”), “feel” (“чувствую”), or “perceive” (“воспринимаю”).

Participants received one worksheet of adjectives and were asked to read the question at the top. Next, they were invited to silently ask themselves the question for each word combination and mark those adjectives that described something they could not imagine. No time limit was placed on responses. After a participant had responded to all of the word combinations on the worksheet, she received the next one (five overall). The experiment lasted 30-40 minutes. The order of the five worksheets was counterbalanced in a Latin square format for different participants. The imageability of adjectives was determined by ranking participants’ responses. A maximal rank (imageability) was assigned to adjectives with the smallest number of participants’ marks. Ranks for adjectives with equal quantity of participants’ marks were assigned according to the principle of “sequential ranks for unique values”.

**Additional categorization of adjectives related to skin sensitivity**

To test the assumption that adjectives related to skin sensations are heterogeneous, we presented an independent sample of 62 native Russian-speaking psychology students (50 women, aged 17 to 33, median age 18) with 120 adjectives related to skin sensitivity (used in Experiment 2 for the mental reactivation of experience) for additional categorization.

Adjectives were printed on a blank in a random order in two columns. On the right of each column of the adjectives there were two blank columns headed with Cyrillic letters “T” and “P”. The letter “T” stood for the first letter of the word “тело” (“body” in English), letter “P” stood for the first letter of the word “рука” (“hand” in English). There were two blank boxes next to each adjective. Instruction was printed at the top of the list: «Прилагательные, написанные ниже, обозначают характеристики предметов, которые мы ощущаем с помощью кожной чувствительности. Для каждой характеристики определите, как Вы её обычно ощущаете. Если Вы ощущаете эту характеристику кожей тела, то поставьте галочку в столбце Т напротив соответствующего прилагательного. Если Вы ощущаете эту характеристику объекта, потрогав его рукой, то поставьте галочку в столбце Р напротив соответствующего прилагательного. Если Вы ощущаете эту характеристику и кожей тела и рукой, то поставьте галочку в двух столбцах напротив соответствующего прилагательного». The English translation is as follows: “The adjectives stated below denote the characteristics of objects perceived by means of skin sensitivity. Specify how you usually perceive each characteristic. If you perceive this characteristic through the skin on your body, tick the box in column “T” near the corresponding adjective. If you perceive this characteristic by touch, tick the box in column “P” near the corresponding adjective. If you perceive this characteristic both by the skin on your body and by touch, tick both boxes in the two columns near the corresponding adjective”. The experiment lasted 20 minutes.

Three types of responses were counted for every adjective: the quantity of isolated ticks in the “body” column, the quantity of isolated ticks in the “hand” column and the quantity of double ticks. Those adjectives with more than 32 isolated ticks in the “hand” column were rated as touch-related (54 adjectives in total, χ^2^ (1, N = 62) = 4.22, p < .05). There was no adjectives with more than 32 isolated ticks in the “body” column. The sum of isolated ticks in the “body” column and double ticks were counted for each adjective. Adjectives with more than 51 such responses overall were rated as related to body skin sensations (17 adjectives in total, χ^2^ (1, N = 62) = 4.05, p < .05). Other adjectives were rated as related to mixed skin sensations (49 adjectives in total) (data are available in the S1 Appendix).
