# Supplementary material for: Mental Reactivation and Pleasantness Judgment of Experience Related to Vision, Hearing, Skin Sensations, Taste and Olfaction
Source: PLoS One. 2016 Jul 11;11(7):e0159036. doi: 10.1371/journal.pone.0159036 (PMC4939968; doi:10.1371/journal.pone.0159036)
Supplement: S1 Table — (DOC) [file pone.0159036.s014.doc]

**S1 Table. Statistical significance of the differences in the median values of the time of the pleasantness judgment of experience predominantly related to different senses (Sense effect, see Fig 9).** The median values of the time of the pleasantness judgment were calculated for each rating on pleasantness; this was done individually for each participant and separately for different types of sense-related experience (see S7 Data). Distributions of the medians of the time of the pleasantness judgment were compared using Wilcoxon signed-rank test (N = 97). Comparisons of the time of the pleasantness judgment of experience related to touch and body skin sensations are not presented in the table because of the absence of statistically significant differences.

| **Direction of differences** | **Pleasantness ratings** | | | | | | |
| --- | --- | --- | --- | --- | --- | --- | --- |
|  | **-3** | **-2** | **-1** | **0** | **+1** | **+2** | **+3** |
| Taste > Olfaction | .423 | .395 | .063 | .368 | .003 | .017 | .111 |
| Hearing > Olfaction | <.001 | .013 | <.001 | <.001 | .009 | .023 | <.001 |
| Vision > Olfaction | <.001 | <.001 | .011 | .009 | <.001 | <.001 | .009 |
| Hearing > Taste | .001 | .127 | <.001 | <.001 | <.001 | <.001 | <.001 |
| Vision > Taste | .008 | .009 | <.001 | <.001 | <.001 | <.001 | .042 |
| Vision > Hearing | .871 | .332 | .999 | .306 | .294 | .070 | .155 |
